# Associations of intermuscular adipose tissue and total muscle wasting score in PG-SGA with low muscle radiodensity and mass in nonmetastatic colorectal cancer: A two-center cohort study

**DOI:** 10.3389/fnut.2022.967902

**Published:** 2022-08-25

**Authors:** Yang Wang, Yuliuming Wang, Guodong Li, Hao Zhang, Hang Yu, Jun Xiang, Zitong Wang, Xia Jiang, Guoqing Yan, Yunxiao Liu, Chunlin Wang, Huan Xiong, Guiyu Wang, Hanping Shi, Ming Liu

**Affiliations:** ^1^Cancer Center, The Second Affiliated Hospital of Harbin Medical University, Harbin, China; ^2^Department of General Surgery, The Fourth Affiliated Hospital of Harbin Medical University, Harbin, China; ^3^Departments of Gastrointestinal Surgery and Department of Clinical Nutrition, Beijing Shijitan Hospital, Capital Medical University, Beijing, China

**Keywords:** low muscle radiodensity, low muscle mass, total muscle wasting score, PG-SGA, intermuscular adipose tissue, nonmetastatic colorectal cancer

## Abstract

**Backgrounds:**

The patient-generated subjective global assessment (PG-SGA) is one of the screening criteria for malnutrition, the skeletal muscle radiodensity (SMD) and skeletal muscle mass index (SMI) are associated with survival in colorectal cancer patients. Body composition parameters can be easily assessed; however, few studies have examined the association between total muscle wasting scores in PG-SGA and body composition parameters and two muscle abnormalities.

**Methods:**

This cohort study included 1,637 stage I-III CRC patients from 2 clinical centers in China, who were enrolled in the training cohort (*n* = 1,005) and validation cohort (*n* = 632). Baseline data were collected prospectively from patients including age, BMI, staging, gait speed, hand grip strength (HGS), peak expiratory flow (PEF), neutrophil-lymphocyte ratio (NLR), intermuscular adipose tissue (IMAT), visceral fat area (VFA) and total muscle wasting score in PG-SGA. Relevant risk factors were subjected to logistic regression analysis and Cox regression analysis to identify characteristics associated with muscle abnormalities and survival. Based on the logistic model results, normograms were established to predict muscle abnormalities, and its discrimination and calibration were assessed using the receiver operating characteristic (ROC) curve and calibration curve. The Kaplan-Meier curves were used to assess the survival of colorectal cancer patients with malnutrition or sarcopenia in an inflammatory state (assessed by NLR).

**Results:**

The mean age of all participants was 57.7 ± 10.6 years (56.9% males) and the prevalence of low SMD and low SMI was 32.2 and 39.5%, respectively. Low SMD rate was significantly associated with age, TNM stage, BMI, IMAT, walking speed, total muscle wasting score and NRS2002 score by logistic regression analysis (*p* < 0.05). Low SMI rate was significantly correlated with age, NLR, BMI, PEF, handgrip strength, calf circumference, walking speed, total muscle wasting score and NRS2002 score (*p* < 0.05). The AUCs of the diagnostic nomograms were 0.859 (95% CI, 0.831–0.886) for low SMD and 0.843 (95% CI, 0.813–0.871) for low SMI in the validation cohort. We also found that patients with colorectal cancer with malnutrition or sarcopenia had a worse prognosis when NLR ≥3.5.

**Conclusion:**

Muscle abnormalities and malnutrition are strongly associated with mortality in patients with non-metastatic colorectal cancer. Early identification and intervention of the associated risk factors may offer new ways to improve patient prognosis.

## Introduction

Skeletal muscle is the organism's effector organ for various simple and complex movements, accounting for about 40% of body weight, and it also plays an important role in the metabolism of carbohydrate, fat and protein (1, 2). Studies have shown that most tumor patients can experience varying degrees of muscle hypofunction and muscle atrophy at different stages of disease development (3), resulting in tumor-associated sarcopenia, which affects the normal metabolism of body components, resulting in higher rates of clinical complications, longer hospital stays, and lower prognosis for survival (4–6).

Globally, colorectal cancer (CRC) accounts for approximately one tenth of diagnosed and fatal cases of malignancy (7). In China, the National Cancer Center has recently reported that colorectal cancer is the second most common malignancy and the fourth most common mortality, and has shown an increasing trend in incidence and mortality since 2000–2016 (8). Cohort studies have shown that the prevalence of sarcopenia in colorectal cancer ranges from ~12–71% (9, 10). The two key components of skeletal muscle loss are quality and quantity, expressed by skeletal muscle radiodensity (SMD) and skeletal muscle mass index (SMI), respectively (11). Computed tomography (CT), long used in cancer diagnosis, is emerging as a cutting-edge strategy for quantifying low SMD and low SMI, while extracting highly accurate body composition data. For example, intermuscular adipose tissue (IMAT) can be obtained by CT (12), but to our knowledge, few studies have explored the association between intermuscular infiltration of excess fat and low SMD and low SMI.

According to the expert consensus of the 2018 Annual Meeting, European Society of Parenteral and Enteral Nutrition (ESPEN) released the Global Leadership Initiative on Malnutrition (GLIM), guidelines stating that reduced muscle mass and low BMI are indicative of malnutrition (13). The PG-SGA also acts as a diagnostic tool for malnutrition and cancer cachexia (14), was developed according to ISPOR principles and is available for download (www.pt-global.org). In this work, we use the term total muscle wasting score to refer to the scored subjective rating of muscle mass in worksheet 4 of the PG-SGA (proposed by FD Ottery et al.), and the study demonstrated that it allows clinicians to make a more visual, graded and dynamic determination of patients' muscle status (15, 16). However, the PG-SGA total muscle wasting score is often overlooked in clinical practice and may be one of the most valid ways to determine low SMD and SMI.

Secondary prevention (i.e., prevention of complications after diagnosis) is one of the key strategies to reduce the heavy burden of colorectal cancer. Low SMD and low SMI are an emerging prognostic factor in colorectal and other cancers (9, 17, 18). Little is known about the risk factors for low SMD and low SMI in CRC. Recognizing and modifying these risk factors may help predict and improve the overall prognosis of colorectal cancer patients. The aim of this study was to investigate the associations of IMAT and total muscle wasting scores in PG-SGA with low SMD and low SMI, and we also comprehensively collected patients' demographic characteristics, hematological parameters, anthropometric measurements, lung function, body composition parameters and nutritional status scores to explore other risk factors associated with low SMD, low SMI, and survival.

Subsequently, we constructed corresponding nomograms and assessed the survival of patients with non-metastatic colorectal cancer under different risk factors.

## Materials and methods

### Study population and setting

**Figure 1:**
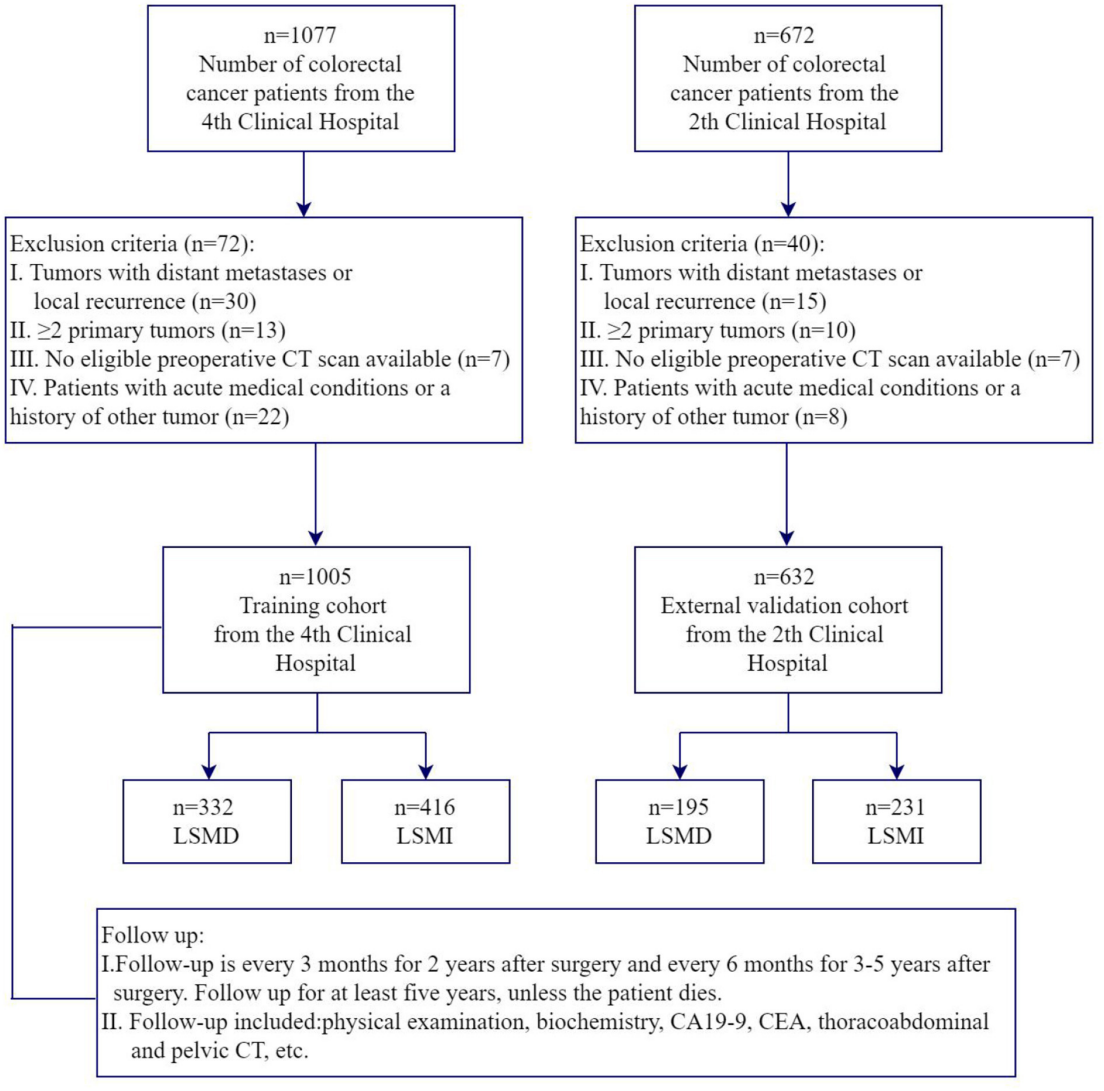
Flowchart of selection of patients.

For our training dataset, we selected patients aged 18–85 years with stage I-III colorectal cancer who underwent radical surgery at the Fourth Hospital of Harbin Medical University from January 2014 to March 2017 for the prospective study. All pre-op patients undergone a standard nutritional status assessment (anthropometric measurements, hematology and nutritional status score, etc.), pulmonary function and abdominal CT scan. Inclusion criteria also included (1) patients with a histological diagnosis of colorectal adenocarcinoma; (2) patients who were conscious, without communication problems, and who agreed to participate in the study. Exclusion criteria include (1) local recurrence or ≥2 primary tumors; (2) no eligible preoperative CT scan available; (3) patients with acute medical conditions or a history of other tumors. For our validation dataset, we selected patients aged 18–85 years with stage I-III colorectal cancer undergoing radical surgery at the Second Hospital of Harbin Medical University from March 2020 to March 2022 with the same admission and exclusion criteria. The primary study outcome was the presence of low SMD and low SMI. The flowchart representing nonmetastatic colorectal cancer patient selection is shown in Figure 1. This study was approved by the ethics committee of the Second Hospital of Harbin Medical University and the Fourth Hospital of Harbin Medical University.

### Data collection

Prospectively collect the following data from patients with non-metastatic colorectal cancer: (1) Demographic characteristics and tumor characteristics: sex, age, diabetes, smoking history (patient who had smoked more than 100 cigarette cumulatively in his or her lifetime) (19), alcohol consumption (patients who have previously drunk alcohol more than once per week), herbal teas with a putative biological effect consumption (patients who have previously drunk tea more than once per week), and weight loss (involuntary weight loss within 1 month), cancer stage; (2) Hematological Biomarkers: creatinine, hemoglobin, prealbumin, serum albumin, neutrophil to lymphocyte ratio (NLR), CRP: C-reactive Protein; (3) Anthropometric measures: body mass index (BMI), handgrip strength (HGS), mid-upper arm circumference (MUAC), triceps skinfold thickness (TSF), mid-arm muscle circumference (MAMC), calf circumference (CC), walking speed; (4) Pulmonary Function: peak expiratory flow (PEF), forced expiratory volume in 1s (FEV1) and vital capacity (VC); (5) Body composition: Intermuscular adipose tissue (IMAT), visceral adipose tissue (VAT) and subcutaneous adipose tissue (SAT); (6) Nutritional status scores: total muscle wasting score in PG-SGA (20), NRS-2002 score (21) and QLQ-C30 score (22). All of these variables (anthropometry and pulmonary function, etc.) were measured by clinicians who were uniformly trained to ensure reproducibility. Relevant data is recorded and stored in an electronic database within 1 week of admission.

Special parameters are measured as follows: (1) Handgrip strength (kg): the patient stands upright with the feet naturally apart and the non-dominant hand grip strength is measured using an electronic grip strength device (EH101; CAMRY). A total of 3 sets are tested, with a 1 min rest after each set, and values are taken to an accuracy of 0.1 kg; (2) MUAC (cm): the physician measures the distance between the surface of the scapula on the dorsal side of the non-dominant arm and the eminence of the elbow, marks the midpoint, asks the patient to drape the upper limb relaxed to the side of the body, wraps the tape measure around the midpoint of the upper arm and ties it tightly, and takes the value to 0.1 cm; (3) TSF (mm): the skin and subcutaneous tissue are pinched up with the thumb and index finger of the left hand at a point 1 cm above the midpoint of the dorsal aspect of the upper arm (from the crest of the shoulder to the midpoint of the ulnar eminence), with the skin fold parallel to the longitudinal axis of the upper arm; the thickness of the skin fold at the midpoint is determined within 3 seconds by the measuring physician with a skin-fold thickness gauge in the right hand, to an accuracy of 0.1 mm; (4) MAMC (cm) = MUAC (cm) −0.314 ^*^ TSF (mm); (5) CC (cm): the patient is seated with the calf at a 90 degree angle to the seat and the left leg is selected for measurement. After exposing the calf, the physician places a tape measure around the thickest part of the calf to measure the circumference and takes the value to 0.1 cm; (6) Walking speed (m/s): the patient walks at the start line at normal speed and the time recorded is from the first foot moving to the first foot over the 6 m finish line. All the above parameters are measured three times, the maximum value is recorded for the step speed and the average value for the other parameters. At the same time, Supplementary Table 1 shows how the total muscle wasting score in PG-SGA is assessed: the score with the highest number of occurrences of the “Muscle Loss Assessment” is counted as the total score for this item. For example, four of the seven muscle scores are 2 and three are 3, giving an overall score of “2.” In our study, the total muscle wasting score in PG-SGA included an assessment of muscle consumption in seven areas: temporalis in the temporal region, deltoid in the clavicular region, deltoid in the shoulder region, interosseous in the hand region, latissimus dorsi, rhomboid and deltoid in the scapular region, quadriceps in the thigh region and gastrocnemius in the calf region.

### SMD, SMI and other body composition parameters

Body composition was measured by diagnostic non-enhanced CT scanning (Somtom Definition Flash, Siemens AG, Erlangen, Germany) prior to radical surgery. Body composition was measured by clinicians who were uniformly trained. Cross-sectional CT images of the third lumbar vertebra (L3) are closely correlated with whole-body adipose and muscle tissue in both cancer patients and healthy populations (23), and it is the de facto gold standard for measuring body composition (muscle and adipose compartments) in oncology (24). The muscles at the L3 level, including the rectus abdominis, internal oblique, external oblique, transverse abdominis, psoas major, psoas square and erector spinae muscles. We selected a single image of the third lumbar vertebra (L3) for body composition quantification, including all skeletal muscle mass, visceral adipose tissue (VAT), intermuscular adipose tissue (IMAT) and subcutaneous adipose tissue (SAT). For adipose tissue, standard CT values range from −190 to −30 Hounsfield Units for IMAT and SAT, and from −150 to −50 Hounsfield Units for VAT; for muscle tissue, the standard CT values range from −29 to 150 Hounsfield Units (HU) (23, 25). We used SliceOmatic Software version 5.0 (TomoVision) to measure tissue area, total abdominal muscle area (TAMA) measured at L3 divided by the square of height as SMI (cm^2^/m^2^), and the mean radiation attenuation value of the muscle group measured at L3 as SMD (HU).

### Definitions of malnutrition and sarcopenia

Malnutrition is defined according to the GLIM criteria, which consists of two modules: phenotypic criteria and etiological criteria. There are 2 possible etiological criteria: (1) reduced food intake or digestive and absorptive function; (2) inflammation or disease burden; And 3 possible phenotypic criteria: (1) weight loss; (2) low BMI; and (3) reduced muscle mass. The NRS2002 was used as the initial screening step as part of GLIM and included into the all patients' routine preoperative assessment. Patients at risk of malnutrition (NRS2002 ≥ 3) were diagnosed as malnourished if they met at least one etiological criterion and one phenotypic criterion at the same time. With regard to the etiological criteria of inflammation, recruitment and activation of inflammatory cells can promote tumor progression, and tumor cells in turn can secrete chemokines, pro-inflammatory cytokines and inflammatory enzymes (26). Therefore, all colorectal cancer patients were considered to have met the etiological criteria by virtue of their diagnosis In our study, we defined low BMI (kg/m^2^) using Asian criteria, with BMI <20 for patients aged over 70 years and <18.5 for those aged <70 years (13). Also, we used the validated value of LSMI to define muscle mass loss.

The diagnosis of sarcopenia is made up of two components: low muscle mass or low muscle quality and low grip strength according to the EWGSOP-2 Asian consensus (11). A diagnosis of sarcopenia is made when 2 criterias are met simultaneously. The cutpoints for low SMD is 32.5 HU in women and 35.5 HU in men (27). Low SMI cutpoints were ≤36.2 and ≤29.6 cm^2^/m^2^ for men and women, respectively (28, 29). In addition, low grip strength is defined as <18 kg for women and <26 kg for men (30).

### Follow-up assessments

We followed up patients at the Fourth Hospital of Harbin Medical University by telephone or in hospital according to National Comprehensive Cancer Network (NCCN) follow-up principles (31). The follow-up visits included physical examination, biochemistry, CA19-9, CEA, abdominal and pelvic ultrasound, thoracoabdominal and pelvic CT or MRI and colonoscopy. Follow-up is every 3 months for 2 years after surgery and every 6 months for 3–5 years after surgery. The last follow-up was in March 2022. Overall survival is calculated from the first day after surgery to the time of death due to any cause.

### Statistical analysis

Statistical analyses of all data were performed using SPSS statistics version 25.0 (IBM, Armonk, NY), R version 4.1.2 (R Project for Statistical Computing, Vienna, Austria), X-tile plots (Yale University School of Medicine, New Haven, Connecticut, USA) and MedCalc software version 20.106 (MedCalc, Mariakerke, Belgium). Continuous data were expressed as means ± SDs and compared using Student's *t*-test. Categorical variables were expressed as frequencies (%) and compared using Pearson's chi-square test or Fisher's exact test. In the training cohort, logistic regression analysis was used to predict low SMD and low SMI, respectively. Significant preoperative factors from the univariate logistic regression analysis (*p* < 0.05) were included in the multivariate logistic regression analysis. Risk factors that proved to be significant in the training cohort were used to create nomograms and the validity of the associated predictive factors was evaluated in the validation cohort. The utility of the developed model was also assessed by calibration curves, decision curve analysis (DCA) and area under the curve (AUC). The calibration and discrimination of the nomogram were assessed using AUC and calibration curves. Also, the net clinical benefit of the nomogram at different threshold probabilities was quantified using DCA (32). In survival analysis, overall survival (OS) was analyzed using standard Cox regression analysis based on the proportional risk assumption. Univariate and multivariate Cox proportional regression was used to analyze preoperative continuous and categorical data, and Kaplan-Meier curves were used to represent survival in patients with low SMD, low SMI, malnutrition, or sarcopenia.

## Results

### Patient characteristics

A total of 1,637 patients were enrolled in the two centers, with 1,005 patients included in the training cohort (the 4th Clinical Hospital) and 632 patients in the validation cohort (the 2nd Clinical Hospital). The baseline characteristics of the patients in terms of demographic, hematological indicators, anthropometric measurements, lung function, body composition parameters and nutritional status scores are shown in Table 1. Males had higher Grip strength, calf circumference, SMD and SMI than females. In the training cohort, the prevalence of low SMD and low SMI was 31.2 and 40.2% in females and 34.3 and 42.2% in males, respectively. In the validation cohort, the prevalence of low SMD and low SMI was 30.0 and 36.6% in females and 31.5 and 36.4% in males, respectively.

**Table 1:** Baseline characteristics of colorectal patients in the two centers^a^.

	**Training cohort**, ***n*** = **1,005**				**External validation cohort**, ***n*** = **632**			
**Characteristics**	**Overall**	**Female** **(*n* = 435)**	**Male** **(*n* = 570)**	***P* value**	**Overall**	**Female** **(*n* = 270)**	**Male** **(*n* = 362)**	***P* value**
**Demographics**								
Age	59.7 ± 10.3	57.7 ± 10.2	61.3 ± 10.1	<0.001	54.4 ± 10.1	53.9 ± 10.2	54.8 ± 10.1	0.226
Diabetes, *n* (%)	177(17.6)	80(18.3)	97(17.0)	0.571	78(12.3)	35(12.9)	43(11.8)	0.682
Alcohol, *n* (%)	165(16.4)	52(11.9)	113(19.8)	0.001	86(13.6)	8(2.9)	78(21.5)	<0.001
Smoking history, *n* (%)	222(22.0)	46(10.5)	176(30.8)	<0.001	121(19.1)	18(6.6)	103(28.4)	<0.001
Tea drinking, *n* (%)	98(9.7)	41(9.4)	57(10.0)	0.761	72(11.3)	30(11.1)	42(11.6)	0.848
**Weight loss**, ***n*** **(%)**				0.106				0.192
Stable	530(52.7)	240(55.2)	290(50.9)		492(77.8)	204(32.3)	288(79.6)	
0–4.9%	354(35.2)	153(35.2)	201(35.3)		124(19.6)	61(9.7)	63(17.4)	
≥5%	121(12.1)	42(9.6)	79(13.9)		16(2.6)	5(0.8)	11(3.0)	
C**ancer stage**, ***n*** **(%)**				<0.001				0.965
I	154(15.3)	68(15.6)	86(15.1)		248(39.2)	106(16.8)	142(39.2)	
II	292(29.1)	158(36.3)	134(23.5)		188(29.7)	79(12.5)	109(17.2)	
III	559(55.6)	209(48.1)	350(61.4)		196(31.1)	85(13.4)	111(30.6)	
**Hematological biomarkers**								
Creatinine, mg/dl	65.1 ± 17.7	61.1 ± 15.8	68.1 ± 18.5	<0.001	76.2 ± 37.8	71.1 ± 49.4	79.9 ± 25.4	0.004
Hemoglobin, g/L	130.3 ± 19.2	128.9 ± 18.7	131.4 ± 19.5	0.041	130.7 ± 23.4	124.9 ± 20.6	135.1 ± 24.4	<0.001
Prealbumin, mg/L	258.2 ± 55.2	260.2 ± 54.8	256.7 ± 55.4	0.318	264.9 ± 51.5	263.4 ± 48.3	266.0 ± 53.7	0.536
Albumin, g/L	44.6 ± 6.3	45.0 ± 6.2	44.3 ± 6.3	0.157	43.3 ± 5.2	43.5 ± 5.3	43.1 ± 5.1	0.330
NLR	3.1 ± 2.1	3.0 ± 1.9	3.2 ± 2.2	0.234	2.5 ± 1.6	2.3 ± 1.2	2.6 ± 1.8	0.031
**CRP**				0.885				0.492
≤10	836(83.2)	475(83.3)	361(83.0)		519(82.1)	294(81.2)	225(83.3)	
>10	169(16.8)	95(16.7)	74(17.0)		113(17.9)	68(18.8)	45(16.7)	
**Human body measurement**								
BMI, kg/m^2^	21.8 ± 4.0	21.2 ± 4.0	21.9 ± 4.1	0.708	23.7 ± 3.6	23.5 ± 3.4	23.7 ± 3.7	0.959
Handgrip strength, kg	22.2 ± 7.1	20.5 ± 5.9	24.4 ± 7.8	<0.001	22.2 ± 9.0	20.4 ± 9.0	23.5 ± 8.8	<0.001
MUAC, cm	23.2 ± 3.6	23.0 ± 3.4	23.4 ± 3.8	0.097	25.4 ± 3.6	24.5 ± 3.5	26.1 ± 3.5	<0.001
TSF, mm	20.6 ± 6.9	21.1 ± 6.8	18.9 ± 6.8	0.042	20.9 ± 7.6	22.2 ± 7.4	19.9 ± 7.6	<0.001
MAMC, cm	16.7 ± 3.9	18.7 ± 3.6	17.0 ± 4.1	0.007	18.8 ± 4.2	17.5 ± 4.0	19.8 ± 4.11	<0.001
CC, cm	30.9 ± 4.7	30.2 ± 4.4	32.0 ± 4.8	<0.001	32.5 ± 4.4	31.9 ± 4.4	32.9 ± 4.3	0.013
Walking speed m/s	1.0 ± 0.6	1.2 ± 0.6	1.1 ± 0.6	0.137	1.1 ± 0.7	1.0 ± 0.6	1.1 ± 0.7	0.570
**Pulmonary function**								
PEF, L/s	4.5 ± 1.3	4.2 ± 1.4	4.7 ± 1.2	<0.001	4.3 ± 1.3	4.2 ± 1.3	4.4 ± 1.3	0.044
FEV1, L	2.0 ± 0.5	1.8 ± 0.4	2.2 ± 0.5	<0.001	1.9 ± 0.4	1.8 ± 0.4	2.0 ± 0.4	<0.001
VC, L	2.7 ± 0.7	2.6 ± 0.7	2.8 ± 0.6	0.232	2.6 ± 0.6	2.4 ± 0.6	2.7 ± 0.5	<0.001
**Body composition**								
IMAT, cm^2^	15.9 ± 6.8	16.1 ± 6.8	15.7 ± 6.7	0.671	14.7 ± 6.4	15.2 ± 6.2	14.2 ± 6.5	0.077
VAT, cm^2^	131.9 ± 54.4	108.5 ± 50.2	149.8 ± 50.5	<0.001	135.3 ± 50.9	115.4 ± 46.1	150.0 ± 49.3	<0.001
SAT, cm^2^	148.7 ± 48.3	171.0 ± 43.5	131.7 ± 44.9	<0.001	147.3 ± 46.2	165.2 ± 42.9	133.8 ± 43.9	<0.001
SMD, HU	36.0 ± 6.1	35.1 ± 5.8	36.6 ± 6.2	<0.001	36.0 ± 6.3	35.6 ± 6.4	36.3 ± 6.3	0.137
SMI, cm^2^/m^2^	44.4 ± 11.8	41.8 ± 12.2	46.5 ± 11.1	<0.001	44.3 ± 11.4	40.5 ± 10.9	47.2 ± 11.0	<0.001
LSMD, *n* (%)	332(33.0)	136(31.2)	196(34.3)	0.297	195(30.8)	81(30.0)	114(31.5)	0.688
LSMI, *n* (%)	416(41.3)	175(40.2)	241(42.2)	0.513	231(36.5)	99(36.6)	132(36.4)	0.958
**Scores**								
**Total muscle wasting score**, ***n*** **(%)**				0.091				0.824
0	337(33.5)	133(30.6)	204(35.8)		345(54.6)	150(55.6)	195(53.8)	
1	207(20.6)	100(23.0)	107(18.8)		98(15.5)	38(14.1)	60(16.5)	
2	251(25.0)	102(23.4)	149(26.1)		100(15.8)	42(15.5)	58(16.0)	
3	210(20.9)	100(23.0)	110(19.3)		89(14.1)	40(14.8)	49(7.7)	
**NRS-2002 score**, ***n*** **(%)**				0.892				0.475
<3	613(61.0)	264(60.7)	349(61.2)		538(85.1)	233(86.3)	305(84.3)	
≥3	392(39.0)	171(39.3)	221(38.8)		94(14.9)	37(13.7)	57(15.7)	
QLQ-C30 score	49.1 ± 14.1	50.4 ± 15.2	48.0 ± 13.1	0.192	50.6 ± 11.3	50.7 ± 11.0	50.5 ± 11.5	0.779

a Values are n (%) or means ± SDs. NLR, neutrophil to lymphocyte ratio; CRP, C-reactive Protein; BMI, body mass index; MUAC, mid-upper arm circumference; TSF, triceps skin fold; MAMC, mid-arm muscle circumference; CC, Calf circumference; PEF, Peak expiratory flow; FEV1, Forced Expiratory Volume In 1s; VC, Vital Capacity; IMAT, Intramuscular adipose tissue; VAT, visceral adipose tissue; SAT, subcutaneous adipose tissue; HU, Hounsfield unit; SMD, skeletal muscle radiodensity; SMI, skeletal muscle index; LSMI, low skeletal muscle mass index; LSMD, low skeletal muscle radiodensity; NRS, nutritional risk screening; QLQ-C30, Quality of Life Questionnare-Core 30. A chi-square test was used for categorical variables to assess differences between groups and Student's t-test or Mann-Whitney U-test was used for continuous variables.

### Predictors associated with low SMD and low SMI

A total of 31 hypothesized risk factors were included in this study. In the training cohort, multivariable logistic regression analysis showed that age, tumor-node-metastasis (TNM) stage, BMI, IMAT, walking speed, total muscle wasting score in PG-SGA and NRS2002 score were significantly associated with low SMD (*P* < 0.05; Table 2); age, NLR, BMI, PEF, handgrip strength, Calf circumference, walking speed, total muscle wasting score in PG-SGA and NRS2002 score were significantly correlated with low SMI (*P* < 0.05; Table 3). We further found that patients with lower age, TNM stage, IMAT, total muscle wasting score in PG-SGA and NRS2002 score had a lower risk of LSMD, while patients with low BMI and low walking speed had a higher risk of LSMD. In addition, patients with lower BMI, PEF, handgrip strength, calf circumference, walking speed had a higher risk of LSMI. Notably, BMI was a strong predictor of low SMD and low SMI, with AUC values of 0.793 and 0.766 in the training cohort, respectively. The best cutpoint for BMI in stratifying LSMI was 18.5 kg/m^2^, similar to the phenotypic criteria for low BMI in the GLIM criteria. As the total muscle wasting score in PG-SGA progressively increases from 0 to 3, the risk of low SMD and low SMI also progressively increases. In terms of body composition parameters, patients with an IMAT >18.6 cm^2^ have a higher risk of low SMD.

**Table 2:** Univariate and multivariate logistic regression analyses of the risk factors associated with low SMD in the training cohort^a^.

	**Univariate analysis**		**Multivariate analysis**	
**Characteristics**	**OR** **(95% CI)**	***P* value**	**OR** **(95% CI)**	***P* value**
**Demographics**				
Sex	0.87(0.67,1.13)	0.297		
Age	1.06(1.04,1.07)	<0.001	1.03(1.02,1.05)	<0.001
Diabetes, *n* (%)	0.87(0.61,1.23)	0.431		
Alcohol, *n* (%)	1.05(0.74,1.49)	0.787		
Smoking history, *n* (%)	1.04(0.76,1.43)	0.788		
Tea drinking, *n* (%)	1.52(1.00,2.33)	0.052		
**Weight loss**, ***n*** **(%)**		<0.001		0.363
Stable	Reference		Reference	
0–4.9%	1.36(1.01,1.82)		1.31(0.87,1.97)	
≥5%	3.29(2.20,4.95)		0.94(0.53,1.69)	
**Cancer stage**, ***n*** **(%)**		<0.001		<0.001
I	Reference		Reference	
II	2.68(1.56,4.62)		2.84(1.41,5.72)	
III	5.08(3.05,8.45)		4.36(2.28,8.33)	
**Hematological biomarkers**				
Creatinine, mg/dl	1.01(1.00,1.01)	0.099		
Hemoglobin, g/L	1.01(1.00,1.01)	0.082		
Prealbumin, mg/L	1.00(0.99,1.00)	0.541		
Albumin, g/L	0.99(0.97,1.01)	0.516		
NLR	1.27(1.17,1.37)	<0.001	1.07(0.97,1.18)	0.176
CRP	1.05(0.90,1.22)	0.080		
**Anthropometric measurements**				
BMI, kg/m^2^	0.74(0.71,0.78)	<0.001	0.92(0.87,0.97)	0.002
Handgrip strength, kg	0.99(0.97,1.01)	0.223		
MUAC, cm	1.00(0.96,1.04)	0.994		
TSF, mm	1.00(0.98,1.02)	0.800		
MAMC, cm	1.00(0.96,1.03)	0.893		
CC, cm	1.01(0.98,1.04)	0.428		
Walking speed, m/s	0.30(0.24,0.39)	<0.001	0.40(0.29,0.54)	<0.001
**Pulmonary function**				
PEF, L/s	1.04(0.94,1.15)	0.459		
FEV1, L	0.89(0.67,1.18)	0.432		
VC, L	0.83(0.68,1.02)	0.071		
**Body composition**				
IMAT, cm^2^	1.06(1.04,1.09)	<0.001	1.10(1.07,1.14)	<0.001
VAT, cm^2^	1.00(0.99,1.01)	0.676		
SAT, cm^2^	0.99(0.99,1.00)	0.268		
VAT/SAT	1.11(0.91,1.35)	0.299		
**Scores**				
**Total muscle wasting score**, ***n*** **(%)**		<0.001		<0.001
0	Reference		Reference	
1	4.50(2.76,7.35)		3.77(2.10,6.76)	
2	10.76(6.81,17.06)		4.37(2.52,7.58)	
3	14.71(9.16,23.63)		7.18(4.03,12.81)	
**NRS-2002 score**, ***n*** **(%)**		<0.001		<0.001
<3	Reference		Reference	
≥3	10.85(7.97,14.77)		5.43(3.43,8.60)	
QLQ-C30 score	1.02(1.01,1.03)	0.002	1.01(0.99,1.02)	0.313

a Data are analyzed by univariate and multivariate logistic regression analysis. Risk factors with significance in univariate analysis were included in the multivariate analysis (p < 0.05). NLR, neutrophil-lymphocyte ratio; CRP, C-reactive Protein; BMI, body mass index; MUAC, mid-upper arm circumference; TSF, triceps skinfold thickness; MAMC, mid-arm muscle circumference; CC, Calf circumference; PEF, Peak expiratory flow; FEV1, Forced Expiratory Volume In 1s; VC, Vital Capacity; IMAT, intermuscular adipose tissue; VAT, visceral adipose tissue; SAT, subcutaneous adipose tissue; HU, Hounsfield unit; SMD, skeletal muscle radiodensity; SMI, skeletal muscle index; LSMI, low skeletal muscle mass index; LSMD, low skeletal muscle radiodensity; NRS, nutritional risk screening; QLQ-C30, Quality of Life Questionnare-Core 30.

**Table 3:** Univariate and multivariate logistic regression analyses of the risk factors associated with low SMI in the training cohort^a^.

	**Univariate analysis**		**Multivariate analysis**	
**Characteristics**	**OR** **(95% CI)**	**P value**	**OR** **(95% CI)**	**P value**
**Demographics**				
Sex	0.92(0.71,1.18)	0.513		
Age	1.09(1.08,1.11)	<0.001	1.07(1.05,1.10)	<0.001
Diabetes, *n* (%)	0.82(0.58,1.14)	0.222		
Alcohol, *n* (%)	0.93(0.66,1.31)	0.691		
Smoking history, *n* (%)	1.05(0.78,1.42)	0.745		
Tea drinking, *n* (%)	1.23(0.81,1.87)	0.339		
Weight loss, *n* (%)		<0.001		0.369
Stable	Reference		Reference	
0–4.9%	1.35(1.03,1.78)		1.12(0.79,1.82)	
≥5%	2.61(1.74,3.91)		0.76(0.39,1.45)	
**Cancer stage**, ***n*** **(%)**		<0.001		0.125
I	Reference		Reference	
II	0.94(0.62,1.41)		0.66(0.35,1.22)	
III	1.83(1.26,2.66)		1.06(0.60,1.85)	
**Hematological biomarkers**				
Creatinine, mg/dl	1.00(0.99,1.01)	0.998		
Hemoglobin, g/L	1.00(1.00,1.01)	0.400		
Prealbumin, mg/L	0.99(0.99,1.01)	0.679		
Albumin, g/L	1.02(1.00,1.04)	0.071		
NLR	1.55(1.41,1.70)	<0.001	1.24(1.12,1.37)	<0.001
CRP	0.99(0.99,1.00)	0.550		
**Anthropometric measurements**				
BMI, kg/m^2^	0.78(0.75,0.82)	<0.001	0.92(0.87,0.97)	0.003
Handgrip strength, kg	0.91(0.89,0.93)	<0.001	0.93(0.90,0.96)	<0.001
MUAC, cm	0.99(0.95,1.02)	0.473		
TSF, mm	0.99(0.98,1.01)	0.461		
MAMC, cm	1.00(0.96,1.03)	0.797		
CC, cm	0.89(0.87,0.92)	<0.001	0.91(0.87,0.95)	<0.001
Walking speed m/s	0.34(0.27,0.42)	<0.001	0.43(0.32,0.58)	<0.001
**Pulmonary function**				
PEF, L/s	0.64(0.57,0.71)	<0.001	0.61(0.53,0.71)	<0.001
FEV1, L	1.15(0.87,1.50)	0.327		
VC, L	0.96(0.79,1.16)	0.679		
**Body composition**				
IMAT, cm^2^	0.99(0.97,1.01)	0.297		
VAT, cm^2^	1.00(1.00,1.01)	0.099		
SAT, cm^2^	0.99(0.99,1.00)	0.596		
VAT/SAT	1.04(0.86,1.26)	0.707		
**Scores**				
**Total muscle wasting score**, ***n*** **(%)**		<0.001		<0.001
0	Reference		Reference	
1	3.3(2.17,5.15)		2.38(1.37,4.13)	
2	11.23(7.46,16.91)		4.91(2.88,8.36)	
3	16.70(10.79,25.86)		8.47(4.81,14.91)	
**NRS-2002 score**, ***n*** **(%)**		<0.001		<0.001
<3	Reference		Reference	
≥3	6.38(4.82,8.45)		2.56(1.56,4.22)	
QLQ-C30 score	1.01(1.00,1.02)	0.003	1.01(0.99,1.02)	0.336

a Data are analyzed by univariate and multivariate logistic regression analysis. Risk factors with significance in univariate analysis were included in the multivariate analysis (p < 0.05). NLR, neutrophil-lymphocyte ratio; CRP, C-reactive Protein; BMI, body mass index; MUAC, mid-upper arm circumference; TSF, triceps skinfold thickness; MAMC, mid-arm muscle circumference; CC, Calf circumference; PEF, Peak expiratory flow; FEV1, Forced Expiratory Volume In 1s; VC, Vital Capacity; IMAT, intermuscular adipose tissue; VAT, visceral adipose tissue; SAT, subcutaneous adipose tissue; HU, Hounsfield unit; SMD, skeletal muscle radiodensity; SMI, skeletal muscle index; LSMI, low skeletal muscle mass index; LSMD, low skeletal muscle radiodensity; NRS, nutritional risk screening; QLQ-C30, Quality of Life Questionnare-Core 30.

### Nomograms construction, validation and clinical performance

**Figure 2:**
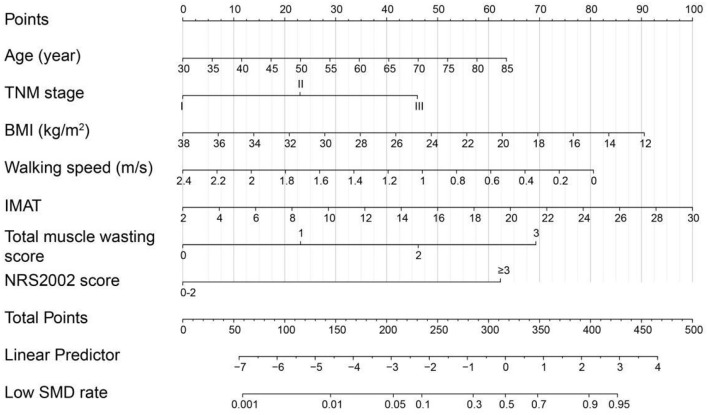
The nomogram for predicting low SMD based on the training cohort (*n* = 1,005).

**Figure 3:**
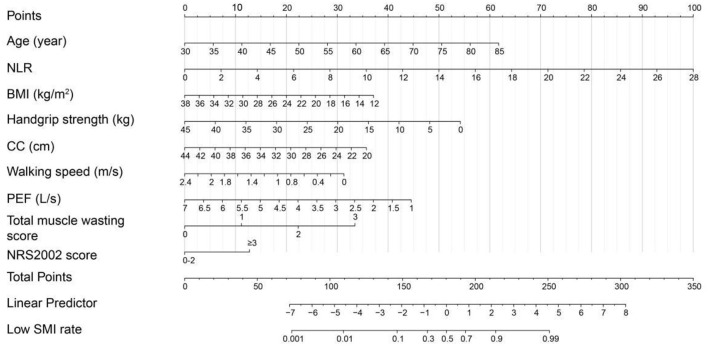
The nomogram for predicting low SMI based on the training cohort (*n* = 1,005).

**Figure 4:**
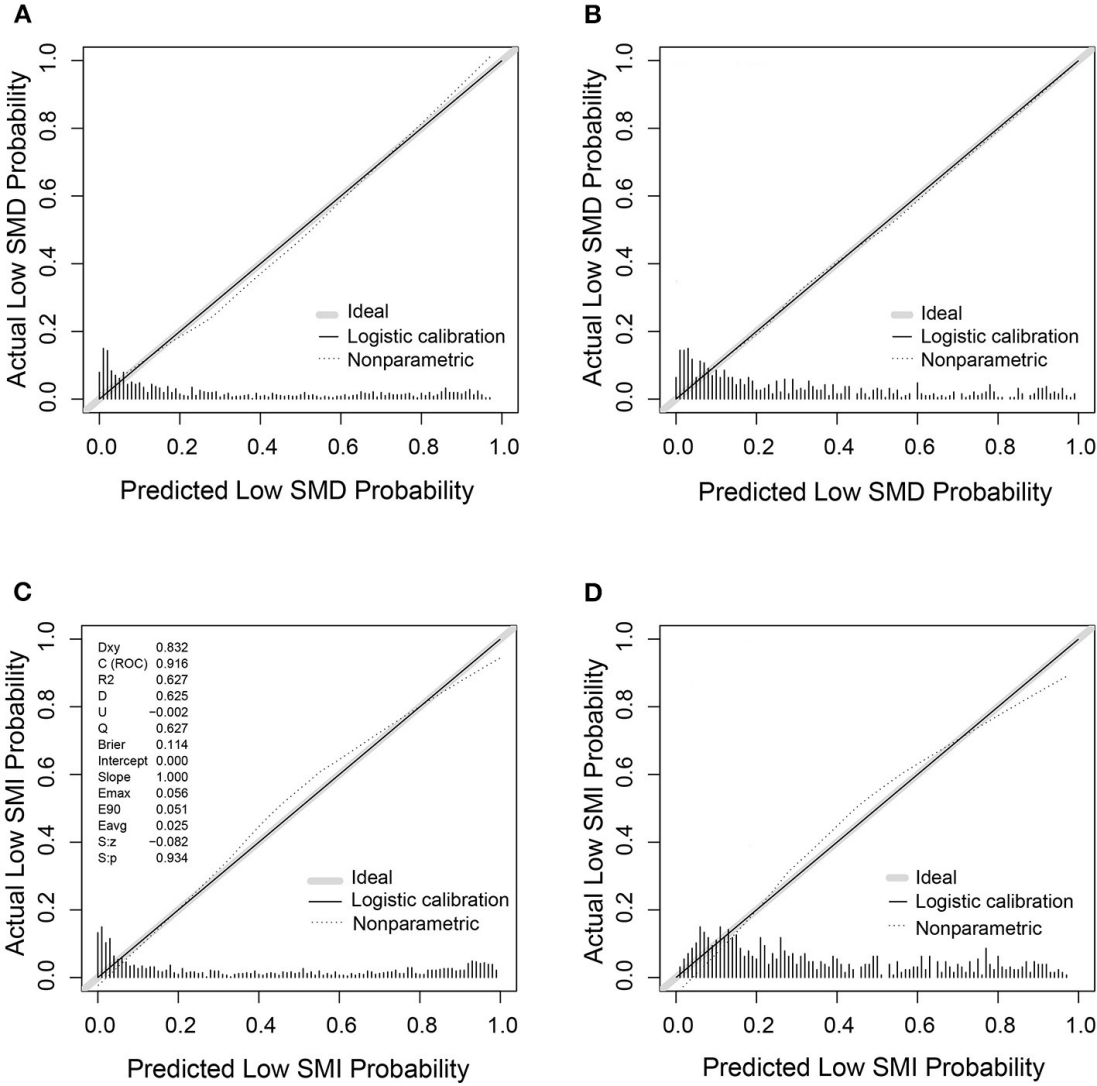
The calibration curves of nomogram for predicting low SMD and low SMI based on the training (*n* = 1,005) and validation (*n* = 632) cohorts. **(A)** The calibration curve for low SMD predictions in the training cohort. **(B)** The calibration curve for low SMD predictions in the validation cohort. **(C)** The calibration curve for low SMI predictions in the training cohort. **(D)** The calibration curve for low SMI predictions in the validation cohort.

**Figure 5:**
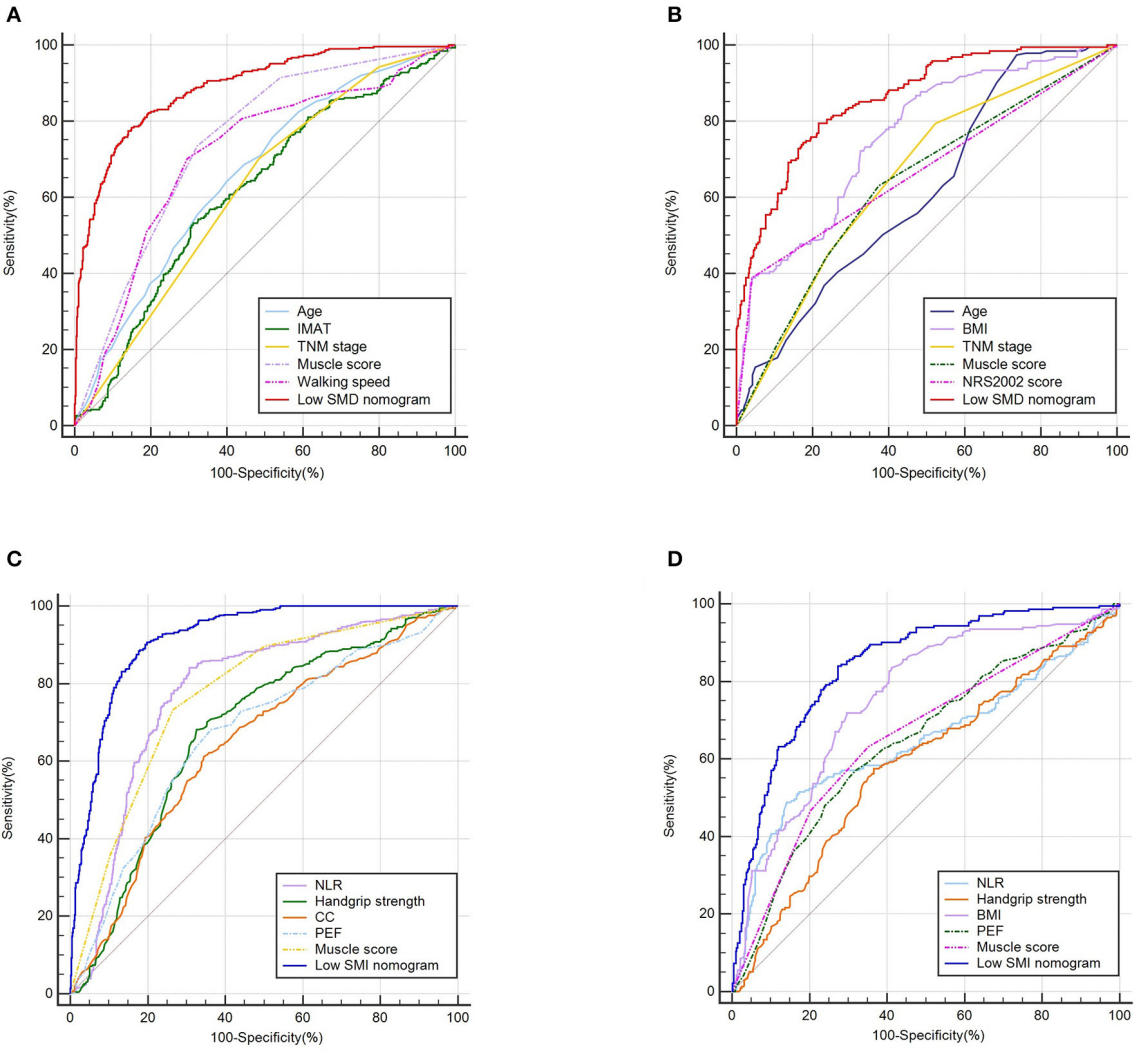
The ROC curves of predictive factors and nomograms for predicting low SMD and low SMI based on the training (*n* = 1,005) and validation (*n* = 632) cohorts. The ROC curves of predictive factors and nomograms for predicting low SMD based on the training **(A)** and validation **(B)** cohorts. The ROC curves of predictive factors and nomograms for predicting low SMI based on the training **(C)** and validation **(D)** cohorts.

Based on the low SMD rate in the training cohort, we constructed a nomogram using seven independent predictors including age, TNM stage, BMI, IMAT, walking speed, total muscle wasting score in PG-SGA and NRS2002 score (Figure 2) after multivariate logistic regression analysis. Similarly, the predictive nomogram containing all the independent risk factors for low SMI in the training cohort is shown in Figure 3. In the validation cohort, we included the above risk factors associated with low SMD and low SMI to demonstrate the validity of the identified risk factors and nomogram. The calibration curve was close to 45 degrees, which indicated that the low SMD and low SMI probabilities predicted by the nomogram in both the training and validation cohorts were in good agreement with the actual probabilities (Figure 4). The DCA curves showed good clinical performance of the two models in diagnosing low SMD and low SMI in both clinical centers (Supplementary Figure 1). To compare the predictive performance of the nomogram with other risk factors, we calculated area under the curve (AUC) (Supplementary Table 2) and plotted the ROC curves for the associated risk factors and nomograms (Figure 5). The AUC values for low SMD and low SMI were 0.890 (95% CI, 0.875 to 0.908) and 0.916 (95% CI, 0.897 to 0.933) in the training cohort and 0.859 (95% CI, 0.831 to 0.886) and 0.843 (95% CI, 0.813 to 0.871) in the validation cohort, respectively.

### Survival analyses

The Kaplan-Meier analysis showed that the 3- and 5-year overall survival rates were 83.2 and 62.8% in the training cohort. A total of 31 preoperative factors were included in the univariate and the multivariate Cox regression analyses. Multivariate Cox proportional risk analysis showed that TNM stage, low SMD and low SMI were significantly associated with OS (*P* < 0.05; Supplementary Table 3). Low SMD [hazard ratio (HR) 2.11, *p* < 0.0001] indicates that patients with LSMD have a 1.11-fold increased risk of death compared to those without LSMD. Low SMI [hazard ratio (HR) 2.31, *p* < 0.0001] indicates that patients with LSMI have a 1.31-fold increased risk of death compared to those without LSMI. Relevant patients were screened according to the criteria of malnutrition and sarcopenia. We found that the prevalence of malnutrition was 33.8% (*n* = 340) and sarcopenia was 34.0% (*n* = 342).

**Figure 6:**
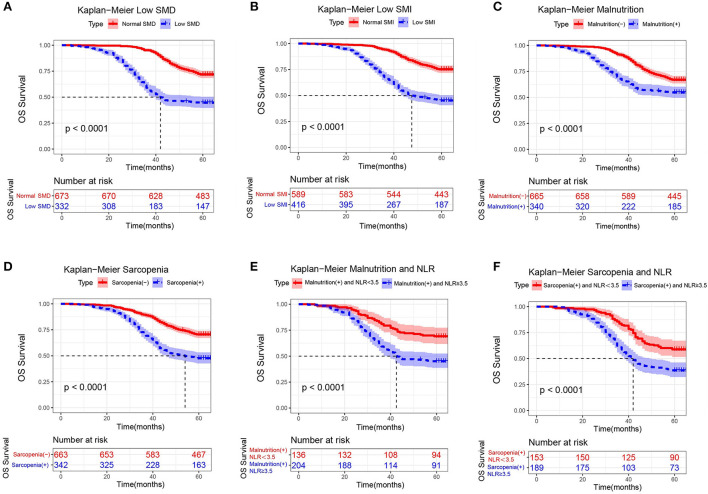
Kaplan-Meier curves for overall survival, stratified by **(A)** low SMD, **(B)** low SMI, **(C)** malnutrition, **(D)** sarcopenia, **(E)** malnutrition and NLR, and **(F)** sarcopenia and NLR in the training cohorts (*n* = 1,005).

As observed in the Kaplan-Meier curve, patients with low SMD (Figure 6A), low SMI (Figure 6B), malnutrition (Figure 6C), or sarcopenia (Figure 6D) had worse survival rates compared to normal patients. We used X-tile to determine that the cut-off value of NLR is 3.5. When only NLR was considered, the KM curve and log-rank test results indicated a significant difference in the distribution of overall survival (OS) between nonmetastatic CRC patients with high NLR (≥3.5) and low NLR (<3.5). Neutrophil-lymphocyte ratio (NLR) is a measure of systemic inflammation, and NLR ≥ 3.5 meets the criteria of moderate to high inflammation. Our study found that patients with NLR ≥ 3.5 and malnutrition had a nearly 1-fold increased risk of death compared to patients with NLR < 3.5 and malnutrition (log-rank *P* < 0.001) (Figure 6E). In addition, a similar presentation was found in patients with sarcopenia (log-rank P < 0.001) (Figure 6F). The study suggests that moderate-to-severe inflammatory status may influence survival in nonmetastatic CRC patients with malnutrition or sarcopenia.

## Discussion

To our knowledge, this is the first study to assess the predictive ability of total muscle wasting score in PG-SGA and intermuscular adipose tissue (IMAT) for CT-derived muscle radiodensity and muscle mass, and the largest study to explore demographic and medical characteristics associated with low SMD and low SMI in colorectal cancer patients based on a Chinese population. We found that as the total muscle wasting risk score in PG-SGA increased from 0 to 3, the risk of low SMD and low SMI also increased, which may be the most direct indicator for clinical assessment of muscle abnormalities. Intermuscular adipose tissue (IMAT), a body composition parameter, better predicted low SMD, but did not correlate clearly with low SMI. When IMAT >18.6 cm^2^, the patient's low SMD rate increased considerably.

To ensure a more comprehensive determination of risk factors for low SMD and low SMI, we collected a total of 31 preoperative risk factors based on six aspects: demographic characteristics, hematological parameters, anthropometry, lung function, body composition parameters and nutritional status score. Our study showed that age, TNM stage, BMI, IMAT, walking speed, total muscle wasting score in PG-SGA and NRS 2002 score were independent factors for low SMD; age, NLR, BMI, PEF, handgrip strength, calf circumference, walking speed, total muscle wasting score in PG-SGA and NRS 2002 score were independent factors for low SMI. The diagnostic nomogram consisting of these preoperative factors successfully predicted low SMD and low SMI in the training cohort and validation cohort with good discrimination and accuracy. It has the potential to help us to identify patients with muscle abnormalities early and to intervene in the early treatment of these patients.

In survival analyses, we found that patients with low SMD, low SMI, malnutrition or sarcopenia had a poorer prognosis. Furthermore, the co-occurrence of malnutrition or sarcopenia and inflammation were associated with a high risk of death, which is consistent with previous results on exploring the coexistence of NLR and sarcopenia in small cell lung cancer (33). One possible explanation is that high NLR is due to a relatively increased neutrophil count, suggesting that the inflammatory state alters the tumor micro-environment, impairing the patient's immune response to malignancy and thereby promoting tumor progression and metastasis (34); it could also be due to a relatively depleted lymphocyte count, which can act as tumor-promoting leucocytes by producing IL-10 and TGF-β, thereby inducing matrix metalloproteinases and regulatory T-cell pathways in the tumor micro-environment (35). NLR, as a marker of systemic inflammation, is not only associated with elevated concentrations of various cytokines in the colorectal cancer circulation (36), but also an enhancer of muscle destruction. For example, a state of systemic inflammation can increase tumor necrosis factor production by tumor or surrounding cells, thereby inhibiting skeletal muscle cell differentiation and promoting muscle atrophy (37, 38).

IMAT is one of the reported measures of myosteatosis (39). Myosteatosis can be understood as a pathological accumulation of fat in muscle, associated with reduced mitochondrial lipid oxidation, insulin resistance and reduced muscle activity (23). Our study showed a direct correlation between IMAT and low SMD, which can be interpreted as an indication that excessive intermuscular infiltration of fat leads to reduced skeletal muscle function as well as reduced CT values of skeletal muscle over a cross-sectional area. We hypothesize that SMD is more influenced by metabolic factors and that high circulating free fatty acid concentrations are thought to impair intermuscular fat metabolism and mitochondrial oxidation, thus leading to fat accumulation into muscle (40). Future studies will be necessary to investigate the exact association between decreased SMD and metabolic disturbances in patients with colorectal cancer.

Muscle atrophy and senescence are important signs of body aging. Increasing age is often accompanied by accelerated muscle loss (41) and redistribution of adipose tissue between or within skeletal muscles (42). The association between old age and muscle abnormalities has also been observed in nonmalignant diseases (43) and other cancers (44). In our training cohort of patients aged ≥60, 62.6% had low SMI and 44.1% had low SMD. Studies have shown that decreases in age-related hormones (e.g., insulin, growth hormone, insulin-like growth factor, testosterone) are strongly associated with the development of sarcopenia (45). As the rapid increase in the world's aging population and elderly cancer patients, the mechanisms of interaction between aging and intramuscular (distributed within muscle tissue) and intermuscular (localized between muscle groups) fat penetration must be explored, and the prevention and treatment of muscle abnormalities has profound implications for improving the quality of life of older people and reducing the burden of diseases such as sarcopenia on society.

Anthropometric (AM) measures such as BMI, walking speed, grip strength (HGS) and calf circumference (CC), MUAC, mid-upper arm circumference (MUAC), triceps fold thickness (TSF), mid-arm muscle circumference (MAMC) may be inexpensive, non-invasive, reproducible, extensive, rapid and simple alternatives to assess sarcopenia, malnutrition, low SMD or low SMI in cancer patients (46). Our findings regarding the association of grip strength (47), calf circumference (48), BMI (49), and walking speed (50) with low muscle mass are consistent with previous studies. After middle age, HGS declines with age at a rate of approximately 1% per year (51). The European guidelines for sarcopenia (11) suggest that grip strength, a supportive measure of sarcopenia, has a significant impact on clinical prognosis in cancer patients. Interestingly, we found that HGS was associated with low SMI but not low SMD, and it is possible that there is no linear association between muscle radiodensity and muscle strength. TSF, one of the components of malnutrition screening, is often used to assess free fat mass. However, we did not find a direct association between TSF and low SMD or low SMI. The study highlights the importance of considering anthropometric parameters simultaneously to provide additional information when assessing risk or planning intervention strategies for these patients.

Sarcopenia not only affects the extremities, but also causes a loss of strength and mass in a wider range of skeletal muscles, including respiratory muscles (e.g., the diaphragm) (52). The decline in respiratory strength associated with aging can also be termed “respiratory sarcopenia” (53). In clinical work, patients often routinely undergo preoperative pulmonary function tests as an important tool to determine the risk of anesthesia. However, few have explored the correlation between pulmonary function indicators and muscle radiodensity and muscle mass. Our study included pulmonary function parameters such as PEF and FEV1, and the results showed a clear association between PEF and low SMI in patients with non-metastatic colorectal cancer compared to FEV1. The reason for this may be that FEV1 is largely confounded by airway obstruction. In contrast, PEF, determined by the strength of the respiratory muscles, is obtained during early expiration prior to airway obstruction (54). Thus, PEF is unaffected by airway obstruction.

Overall, the nomogram allows us to assess the probability of patients developing LSMI and LSMD based on the scores of each relevant factor, so that we can carry out nutritional interventions for such risk groups, which is of great importance for clinical work. However, the current study has some limitations worth noting. First, the mean BMI in our study was low and may be more representative of the Chinese population than other populations. Further studies are needed to validate our findings in overweight or class I-III obese populations. Secondly, selection bias may affect the generalisability of the results as this study only included patients with curable colorectal cancer. Therefore, the lack of data on patients with metastatic colorectal cancer may limit the scope of its application. Third, even with relatively large cohorts, more data is needed to determine cut-off values for these predictors so that clinicians could be given definitive guidance in determining low SMD and low SMI. Finally, because it is a cross-sectional study and it could not determine the causality between low SMI/SMD and enrolled factors, which should be deemed as one of the limitations in the present research.

## Conclusion

In conclusion, our study is the first to demonstrate the predictive value of total muscle wasting score in PG-SGA and intermuscular adipose tissue (IMAT) for muscle abnormalities. The study also shows that demographic characteristics combined with nutrition-related medical parameters can provide a more comprehensive risk assessment of low SMD and low SMI in patients with non-metastatic colorectal cancer. These two distinct muscle abnormalities suggest different biological mechanisms of fat penetration and muscle failure, which may explain why low SMD and low SMI uniquely affect patient prognosis. Furthermore, we found that patients with malnutrition or sarcopenia in a systemic inflammatory state were at higher risk of death. Future exploration of the mechanisms of muscle abnormalities and inflammatory states may provide new directions for clinical intervention.

## Data availability statement

The original contributions presented in the study are included in the article/Supplementary material, further inquiries can be directed to the corresponding author/s.

## Ethics statement

This study was reviewed and approved by the Research Ethics Committee of the Second Affiliated Hospital of Harbin Medical University and the Fourth Affiliated Hospital of Harbin Medical University, China. Patients/participants provided written informed consent to participate in this study.

## Author contributions

YaW designed the study, contributed to project conception, performed the statistical analysis, and wrote the paper. YuW and GL contributed to project conception, data analysis, interpretation, editing, and critical review. HZ and HY contributed to study design, interpretation, and editing. JX and ZW conducted the literature search and collected the data. XJ and GY collected the data and contributed to analysis and editing. YL, CW, and HX contributed to interpretation and editing. HS and GW contributed to editing and critical review. ML contributed to project conception, interpreted the results, development of the overall research plan, data analysis, interpretation, editing, and critical review. All authors read and approved the final version submitted.

## Funding

This project was supported by the Natural Science Foundation of Heilongjiang Province, China-Joint Guidance Project (Grant No. LH2020H066).

## Conflict of interest

The authors declare that the research was conducted in the absence of any commercial or financial relationships that could be construed as a potential conflict of interest.

## Publisher's note

All claims expressed in this article are solely those of the authors and do not necessarily represent those of their affiliated organizations, or those of the publisher, the editors and the reviewers. Any product that may be evaluated in this article, or claim that may be made by its manufacturer, is not guaranteed or endorsed by the publisher.

## Supplementary material

Supplementary Figure 1 Decision curve analysis (DCA) of the nomogram for predicting low SMD and Low SMI based on the training (*n* = 1,005) and validation (*n* = 632) cohorts. **(A)** The DCA for low SMD predictions in the training cohort. **(B)** The DCA for low SMD predictions in the validation cohort. **(C)** The DCA for low SMI predictions in the training cohort. **(D)** The DCA for low SMI predictions in the validation cohort.

Supplementary Table 1 Assessment of muscle wasting.

Supplementary Table 2 Area under the curve (AUC) for preoperative predictive factors in the training and validation cohort.

Supplementary Table 3 Univariate and multivariate cox regression analyses for overall survival (OS) in the training cohort.

## Abbreviations

PG-SGA: patient-generated subjective global assessment
CRC: colorectal cancer
CT: computed tomography
ESPEN: European Society of Parenteral and Enteral Nutrition
GLIM: Global Leadership Initiative on Malnutrition
EWGSOP-2: European Working Group on Sarcopenia in Older People 2
NCCN: National Comprehensive Cancer Network
NLR: neutrophil-lymphocyte ratio
CRP: C-reactive Protein
BMI: body mass index
TNM: tumor-node-metastasis
MUAC: mid-upper arm circumference
TSF: triceps skinfold thickness
MAMC: mid-arm muscle circumference
CC: Calf circumference
PEF: Peak expiratory flow
FEV1: Forced Expiratory Volume In 1s
VC: Vital Capacity
IMAT: intermuscular adipose tissue
VAT: visceral adipose tissue
SAT: subcutaneous adipose tissue
L3: third lumbar vertebra
DCA: decision curve analysis
AUC: area under the curve
ROC: receiver-operating characteristic
HU: Hounsfield unit
SMD: skeletal muscle radiodensity
SMI: skeletal muscle index
LSMI: low skeletal muscle mass index
LSMD: low skeletal muscle radiodensity
NRS: nutritional risk screening
QLQ-C30: Quality of Life Questionnare-Core 30.

## References

[1] Frontera WR Ochala J. Skeletal muscle: a brief review of structure and function. Calcif Tissue Int. (2015) 96:183–95. 10.1007/s00223-014-9915-y 25294644

[2] Larsson L Degens H Li M Salviati L Lee YI Thompson W. Sarcopenia: aging-related loss of muscle mass and function. Physiol Rev. (2019) 99:427–511. 10.1152/physrev.00061.2017 30427277 PMC6442923

[3] Kazemi-Bajestani SM Mazurak VC Baracos V. Computed tomography-defined muscle and fat wasting are associated with cancer clinical outcomes. Semin Cell Dev Biol. (2016) 54:2–10. 10.1016/j.semcdb.2015.09.001 26343952

[4] Barret M Antoun S Dalban C Malka D Mansourbakht T Zaanan A. Sarcopenia is linked to treatment toxicity in patients with metastatic colorectal cancer. Nutr Cancer. (2014) 66:583–9. 10.1080/01635581.2014.894103 24707897

[5] Prado CM Baracos VE McCargar LJ Reiman T Mourtzakis M Tonkin K. Sarcopenia as a determinant of chemotherapy toxicity and time to tumor progression in metastatic breast cancer patients receiving capecitabine treatment. Clin Cancer Res. (2009) 15:2920–6. 10.1158/1078-0432.CCR-08-2242 19351764

[6] Prado CM Cushen SJ Orsso CE Ryan AM. Sarcopenia and cachexia in the era of obesity: clinical and nutritional impact. Proc Nutr Soc. (2016) 75:188–98. 10.1017/S0029665115004279 26743210

[7] Sung H Ferlay J Siegel RL Laversanne M Soerjomataram I Jemal A. Global cancer statistics 2020: GLOBOCAN estimates of incidence and mortality worldwide for 36 cancers in 185 countries. CA Cancer J Clin. (2021) 71:209–49. 10.3322/caac.21660 33538338

[8] Zheng R Zhang S Zeng H Wang S Sun K Chen R. Cancer incidence and mortality in China, 2016. J Natl Cancer Inst. (2022) 5:38. 10.1016/j.jncc.2022.02.002 PMC11256658 39035212

[9] Malietzis G Currie AC Athanasiou T Johns N Anyamene N Glynne-Jones R. Influence of body composition profile on outcomes following colorectal cancer surgery. Br J Surg. (2016) 103:572–80. 10.1002/bjs.10075 26994716

[10] Pedziwiatr M Pisarska M Major P Grochowska A Matlok M Przeczek K. Laparoscopic colorectal cancer surgery combined with enhanced recovery after surgery protocol (ERAS) reduces the negative impact of sarcopenia on short-term outcomes. Eur J Surg Oncol. (2016) 42:779–87. 10.1016/j.ejso.2016.03.037 27156809

[11] Cruz-Jentoft AJ Bahat G Bauer J Boirie Y Bruyere O Cederholm T. Sarcopenia: revised European consensus on definition and diagnosis. Age Ageing. (2019) 48:601. 10.1093/ageing/afz046 31081853 PMC6593317

[12] Muresan BT Sanchez Juan C Artero A Hernandez Machancoses A Almendros-Blanco P Montoro A. Measurement of body composition in cancer patients using CT planning scan at the third lumbar vertebra. Nutr Hosp. (2019) 36:1307–14. 10.20960/nh.2435 31718205

[13] Cederholm T Jensen GL Correia M Gonzalez MC Fukushima R Higashiguchi T. GLIM criteria for the diagnosis of malnutrition - A consensus report from the global clinical nutrition community. Clin Nutr. (2019) 38:1–9. 10.1016/j.clnu.2018.08.002 30181091

[14] De Groot LM Lee G Ackerie A van der Meij BS. Malnutrition Screening and Assessment in the Cancer Care Ambulatory Setting: Mortality Predictability and Validity of the Patient-Generated Subjective Global Assessment Short form (PG-SGA SF) and the GLIM Criteria. Nutrients. (2020) 12:2287. 10.3390/nu12082287 32751724 PMC7468976

[15] Jager-Wittenaar H Ottery FD. Assessing nutritional status in cancer: role of the Patient-Generated Subjective Global Assessment. Curr Opin Clin Nutr Metab Care. (2017) 20:322–9. 10.1097/MCO.0000000000000389 28562490

[16] Erickson N Storck LJ Kolm A Norman K Fey T Schiffler V. Tri-country translation, cultural adaptation, and validity confirmation of the Scored Patient-generated subjective global assessment. Support Care Cancer. (2019) 27:3499–507. 10.1007/s00520-019-4637-3 30684046

[17] Martin L Birdsell L Macdonald N Reiman T Clandinin MT McCargar LJ. Cancer cachexia in the age of obesity: skeletal muscle depletion is a powerful prognostic factor, independent of body mass index. J Clin Oncol. (2013) 31:1539–47. 10.1200/JCO.2012.45.2722 23530101

[18] Cespedes Feliciano EM Avrutin E Caan BJ Boroian A Mourtzakis M. Screening for low muscularity in colorectal cancer patients: a valid, clinic-friendly approach that predicts mortality. J Cachexia Sarcopenia Muscle. (2018) 9:898–908. 10.1002/jcsm.12317 30066490 PMC6204585

[19] Foxon F Selya AS. Electronic cigarettes, nicotine use trends and use initiation ages among US adolescents from 1999 to 2018. Addiction. (2020) 115:2369–78. 10.1111/add.15099 32335976 PMC7606254

[20] Bauer J Capra S Ferguson M. Use of the scored patient-generated subjective global assessment (PG-SGA) as a nutrition assessment tool in patients with cancer. Eur J Clin Nutr. (2002) 56:779–85. 10.1038/sj.ejcn.1601412 12122555

[21] Kondrup J Allison SP Elia M Vellas B Plauth M Educational and Clinical Practice Committee. ESPEN guidelines for nutrition screening 2002. Clin Nutr. (2003) 22:415–21. 10.1016/S0261-5614(03)00098-0 12880610

[22] Aaronson NK Ahmedzai S Bergman B Bullinger M Cull A Duez NJ. The European organization for research and treatment of cancer QLQ-C30: a quality-of-life instrument for use in international clinical trials in oncology. J Natl Cancer Inst. (1993) 85:365–76. 10.1093/jnci/85.5.365 8433390

[23] Mourtzakis M Prado CM Lieffers JR Reiman T McCargar LJ Baracos VE. practical and precise approach to quantification of body composition in cancer patients using computed tomography images acquired during routine care. Appl Physiol Nutr Metab. (2008) 33:997–1006. 10.1139/H08-075 18923576

[24] van Vugt JL Levolger S Gharbharan A Koek M Niessen WJ Burger JW. A comparative study of software programmes for cross-sectional skeletal muscle and adipose tissue measurements on abdominal computed tomography scans of rectal cancer patients. J Cachexia Sarcopenia Muscle. (2017) 8:285–97. 10.1002/jcsm.12158 27897414 PMC5697014

[25] Yip C Dinkel C Mahajan A Siddique M Cook GJ Goh V. Imaging body composition in cancer patients: visceral obesity, sarcopenia and sarcopenic obesity may impact on clinical outcome. Insights Imaging. (2015) 6:489–97. 10.1007/s13244-015-0414-0 26070723 PMC4519815

[26] Singh N Baby D Rajguru JP Patil PB Thakkannavar SS Pujari VB. Inflammation and cancer. Ann Afr Med. (2019) 18:121–6. 10.4103/aam.aam_56_18 31417011 PMC6704802

[27] Kroenke CH Prado CM Meyerhardt JA Weltzien EK Xiao J Cespedes Feliciano EM. Muscle radiodensity and mortality in patients with colorectal cancer. Cancer. (2018) 124:3008–15. 10.1002/cncr.31405 29797673 PMC6033621

[28] Fujiwara N Nakagawa H Kudo Y Tateishi R Taguri M Watadani T. Sarcopenia, intramuscular fat deposition, and visceral adiposity independently predict the outcomes of hepatocellular carcinoma. J Hepatol. (2015) 63:131–40. 10.1016/j.jhep.2015.02.031 25724366

[29] Mardian Y Yano Y Ratnasari N Choridah L Wasityastuti W Setyawan NH. Sarcopenia and intramuscular fat deposition are associated with poor survival in Indonesian patients with hepatocellular carcinoma: a retrospective study. BMC Gastroenterol. (2019) 19:229. 10.1186/s12876-019-1152-4 31888500 PMC6937974

[30] Chen LK Liu LK Woo J Assantachai P Auyeung TW Bahyah KS. Sarcopenia in Asia: consensus report of the Asian Working Group for Sarcopenia. J Am Med Dir Assoc. (2014) 15:95–101. 10.1016/j.jamda.2013.11.025 24461239

[31] Benson AB Venook AP Al-Hawary MM Cederquist L Chen YJ Ciombor KK. NCCN Guidelines Insights: Colon Cancer, Version 2.2018. J Natl Compr Canc Netw. (2018) 16:359–69. 10.6004/jnccn.2018.0021 29632055 PMC10184502

[32] Iasonos A Schrag D Raj GV Panageas KS. How to build and interpret a nomogram for cancer prognosis. J Clin Oncol. (2008) 26:1364–70. 10.1200/JCO.2007.12.9791 18323559

[33] Go SI Park MJ Song HN Kang MH Park HJ Jeon KN. Sarcopenia and inflammation are independent predictors of survival in male patients newly diagnosed with small cell lung cancer. Support Care Cancer. (2016) 24:2075–84. 10.1007/s00520-015-2997-x 26546456

[34] Shu Y Qin M Song Y Tang Q Huang Y Shen P. M2 polarization of tumor-associated macrophages is dependent on integrin beta3 via peroxisome proliferator-activated receptor-gamma up-regulation in breast cancer. Immunology. (2020) 160:345–56. 10.1111/imm.13196 32311768 PMC7370165

[35] Grenader T Nash S Plotkin Y Furuse J Mizuno N Okusaka T. Derived neutrophil lymphocyte ratio may predict benefit from cisplatin in the advanced biliary cancer: the ABC-02 and BT-22 studies. Ann Oncol. (2015) 26:1910–6. 10.1093/annonc/mdv253 26037798

[36] Kantola T Klintrup K Vayrynen JP Vornanen J Bloigu R Karhu T. Stage-dependent alterations of the serum cytokine pattern in colorectal carcinoma. Br J Cancer. (2012) 107:1729–36. 10.1038/bjc.2012.456 23059742 PMC3493870

[37] Guttridge DC Mayo MW Madrid LV Wang CY Baldwin AS Jr. NF-kappaB-induced loss of MyoD messenger RNA: possible role in muscle decay and cachexia. Science. (2000) 289:2363–6. 10.1126/science.289.5488.2363 11009425

[38] Fearon KC Glass DJ Guttridge DC. Cancer cachexia: mediators, signaling, and metabolic pathways. Cell Metab. (2012) 16:153–66. 10.1016/j.cmet.2012.06.011 22795476

[39] Goodpaster BH Bergman BC Clark RV Goodpaster BH Bergman BC Clark RV. Myosteatosis in the context of skeletal muscle function deficit: an interdisciplinary workshop at the national institute on aging. Front Physiol. (2020) 11:963. 10.3389/fphys.2020.00963 32903666 PMC7438777

[40] Miljkovic I Zmuda JM. Epidemiology of myosteatosis. Curr Opin Clin Nutr Metab Care. (2010) 13:260–4. 10.1097/MCO.0b013e328337d826 20179586 PMC2872135

[41] Delmonico MJ Harris TB Lee JS Visser M Nevitt M Kritchevsky SB. Alternative definitions of sarcopenia, lower extremity performance, and functional impairment with aging in older men and women. J Am Geriatr Soc. (2007) 55:769–74. 10.1111/j.1532-5415.2007.01140.x 17493199

[42] Stout MB Justice JN Nicklas BJ Kirkland JL. Physiological aging: links among adipose tissue dysfunction, diabetes, and frailty. Physiology. (2017) 32:9–19. 10.1152/physiol.00012.2016 27927801 PMC5338596

[43] Stangl MK Bocker W Chubanov V Ferrari U Fischereder M Gudermann T. Sarcopenia-endocrinological and neurological aspects. Exp Clin Endocrinol Diabetes. (2019) 127:8–22. 10.1055/a-0672-1007 30199918

[44] Weinberg MS Shachar SS Muss HB Deal AM Popuri K Yu H. Beyond sarcopenia: characterization and integration of skeletal muscle quantity and radiodensity in a curable breast cancer population. Breast J. (2018) 24:278–84. 10.1111/tbj.12952 29139618 PMC6414810

[45] Janssen I Baumgartner RN Ross R Rosenberg IH Roubenoff R. Skeletal muscle cutpoints associated with elevated physical disability risk in older men and women. Am J Epidemiol. (2004) 159:413–21. 10.1093/aje/kwh058 14769646

[46] Cao J Xu H Li W Guo Z Lin Y Shi Y. Nutritional assessment and risk factors associated to malnutrition in patients with esophageal cancer. Curr Probl Cancer. (2021) 45:100638. 10.1016/j.currproblcancer.2020.100638 32829957

[47] Moreau J Ordan MA Barbe C Mazza C Perrier M Botsen D. Correlation between muscle mass and handgrip strength in digestive cancer patients undergoing chemotherapy. Cancer Med. (2019) 8:3677–84. 10.1002/cam4.2238 31115188 PMC6639177

[48] Souza NC Gonzalez MC Martucci RB Rodrigues VD de Pinho NB Qureshi AR. Comparative analysis between computed tomography and surrogate methods to detect low muscle mass among colorectal cancer patients. JPEN J Parenter Enteral Nutr. (2020) 44:1328–37. 10.1002/jpen.1741 31736112

[49] Iannuzzi-Sucich M Prestwood KM Kenny AM. Prevalence of sarcopenia and predictors of skeletal muscle mass in healthy, older men and women. J Gerontol A Biol Sci Med Sci. (2002) 57:M772–7. 10.1093/gerona/57.12.M772 12456735

[50] Beavers KM Beavers DP Houston DK Harris TB Hue TF Koster A. Associations between body composition and gait-speed decline: results from the health, aging, and body composition study. Am J Clin Nutr. (2013) 97:552–60. 10.3945/ajcn.112.047860 23364001 PMC3578402

[51] Rantanen T Masaki K Foley D Izmirlian G White L Guralnik JM. Grip strength changes over 27 yr in Japanese-American men. J Appl Physiol. (1985) 85:2047–53. 10.1152/jappl.1998.85.6.2047 9843525

[52] Ro HJ Kim DK Lee SY Seo KM Kang SH Suh HC. Relationship between respiratory muscle strength and conventional sarcopenic indices in young adults: a preliminary study. Ann Rehabil Med. (2015) 39:880–7. 10.5535/arm.2015.39.6.880 26798601 PMC4720763

[53] Kera T Kawai H Hirano H Kojima M Watanabe Y Motokawa K. Definition of respiratory sarcopenia with peak expiratory flow rate. J Am Med Dir Assoc. (2019) 20:1021–5. 10.1016/j.jamda.2018.12.013 30737167

[54] Kera T Kawai H Hirano H Kojima M Fujiwara Y Ihara K. Relationships among peak expiratory flow rate, body composition, physical function, and sarcopenia in community-dwelling older adults. Aging Clin Exp Res. (2018) 30:331–40. 10.1007/s40520-017-0777-9 28560545

