# Supplementary figures and images for: Associations of intermuscular adipose tissue and total muscle wasting score in PG-SGA with low muscle radiodensity and mass in nonmetastatic colorectal cancer: A two-center cohort study
Source: Front Nutr. 2022 Aug 25;9:967902. doi: 10.3389/fnut.2022.967902 (PMC9452825; doi:10.3389/fnut.2022.967902)

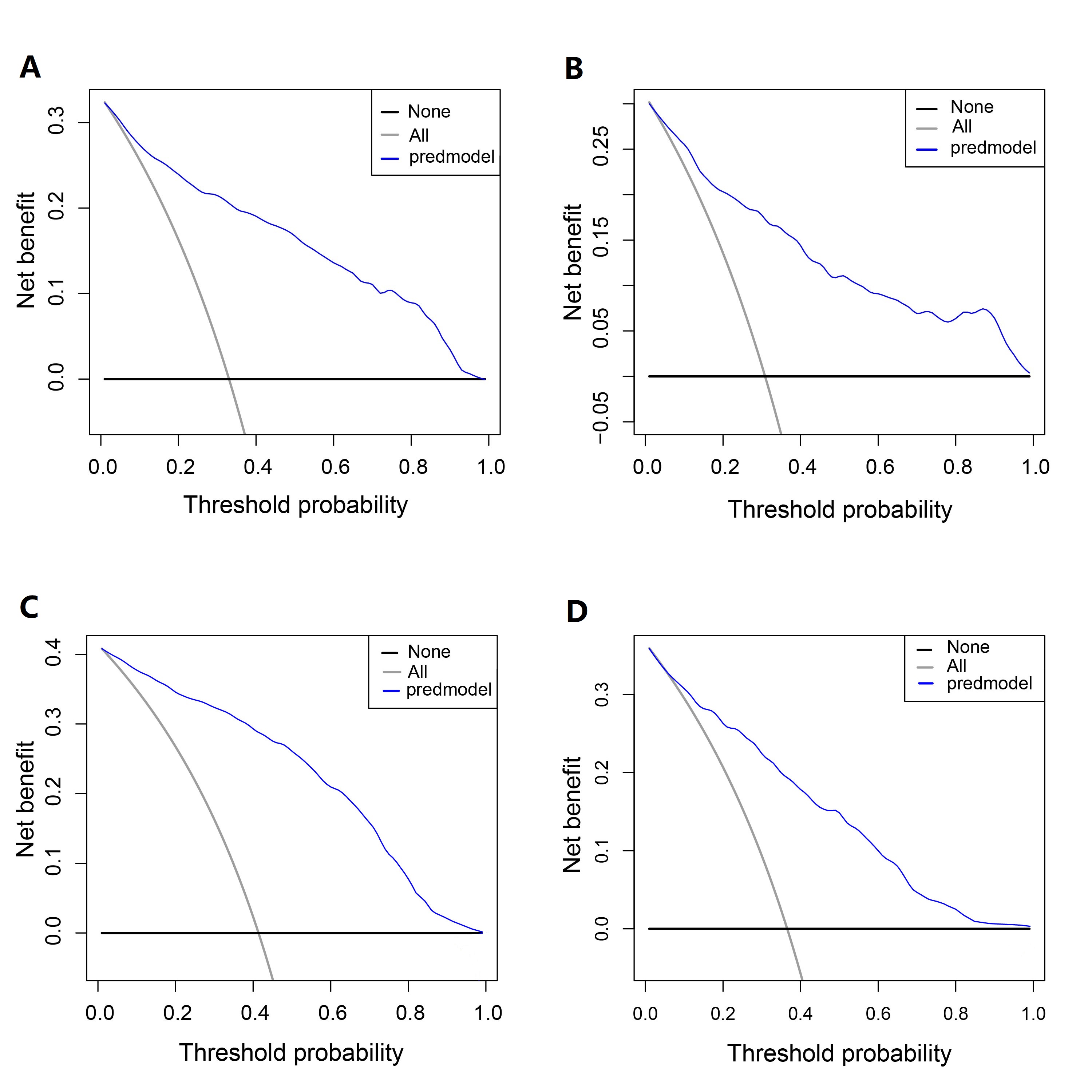

Supplement: Supplementary Figure 1 — Decision curve analysis (DCA) of the nomogram for predicting low SMD and Low SMI based on the training (n = 1,005) and validation (n = 632) cohorts. (A) The DCA for low SMD predictions in the training cohort. (B) The DCA for low SMD predictions in the validation cohort. (C) The DCA for low SMI predictions in the training cohort. (D) The DCA for low SMI predictions in the validation cohort. [file Image_1.JPEG]
