# Supplementary material for: Associations of intermuscular adipose tissue and total muscle wasting score in PG-SGA with low muscle radiodensity and mass in nonmetastatic colorectal cancer: A two-center cohort study
Source: Front Nutr. 2022 Aug 25;9:967902. doi: 10.3389/fnut.2022.967902 (PMC9452825; doi:10.3389/fnut.2022.967902)
Supplement: Supplementary Table 1 — Assessment of muscle wasting. [file Table_1.docx]

**Supplementary Table 1 | Assessment of muscle wasting^1^**

| Muscle | Check the key points | 0 points | 1 points | 2 points | 3 points |
| --- | --- | --- | --- | --- | --- |
| Temporal (temporalis muscle) | Direct observation, with the patient's head turned to one side | No visible depressions | Mild depression | Depressions | Significant depression |
|  |  | 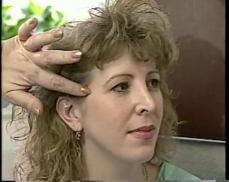 | 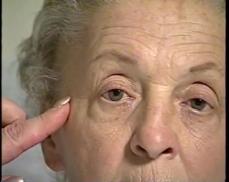 | 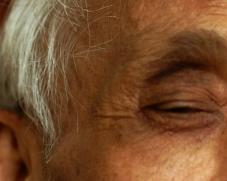 | 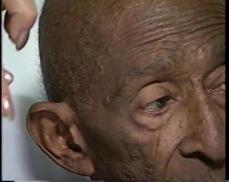 |
| Clavicle area (chest deltoid) | Look for protruding collarbones | Clavicle not visible in men, visible but not protruding in women | Partial projection | Protruding | clearly highlighted |
|  |  | 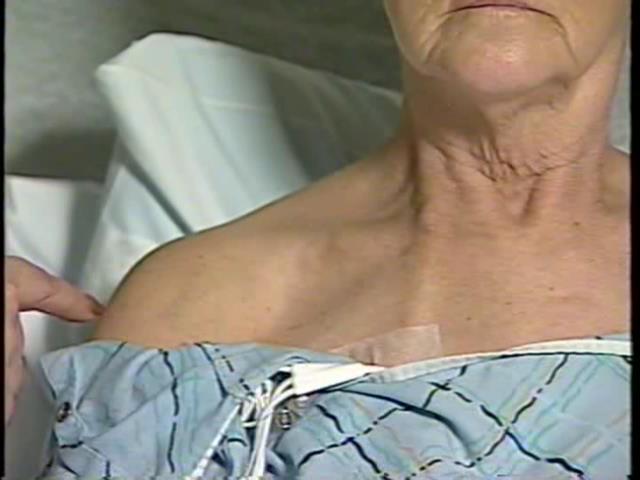 | 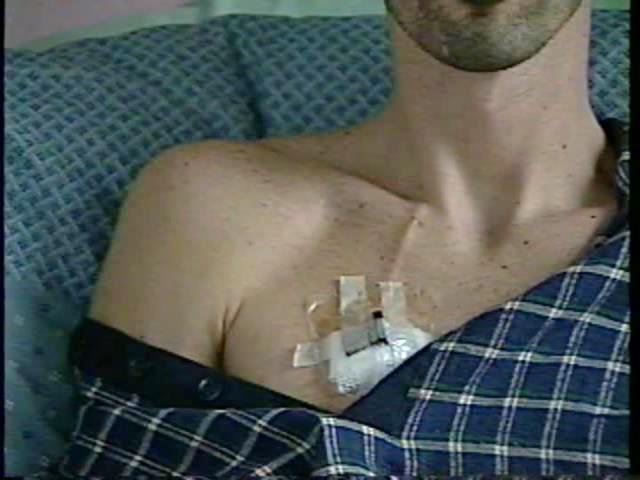 | 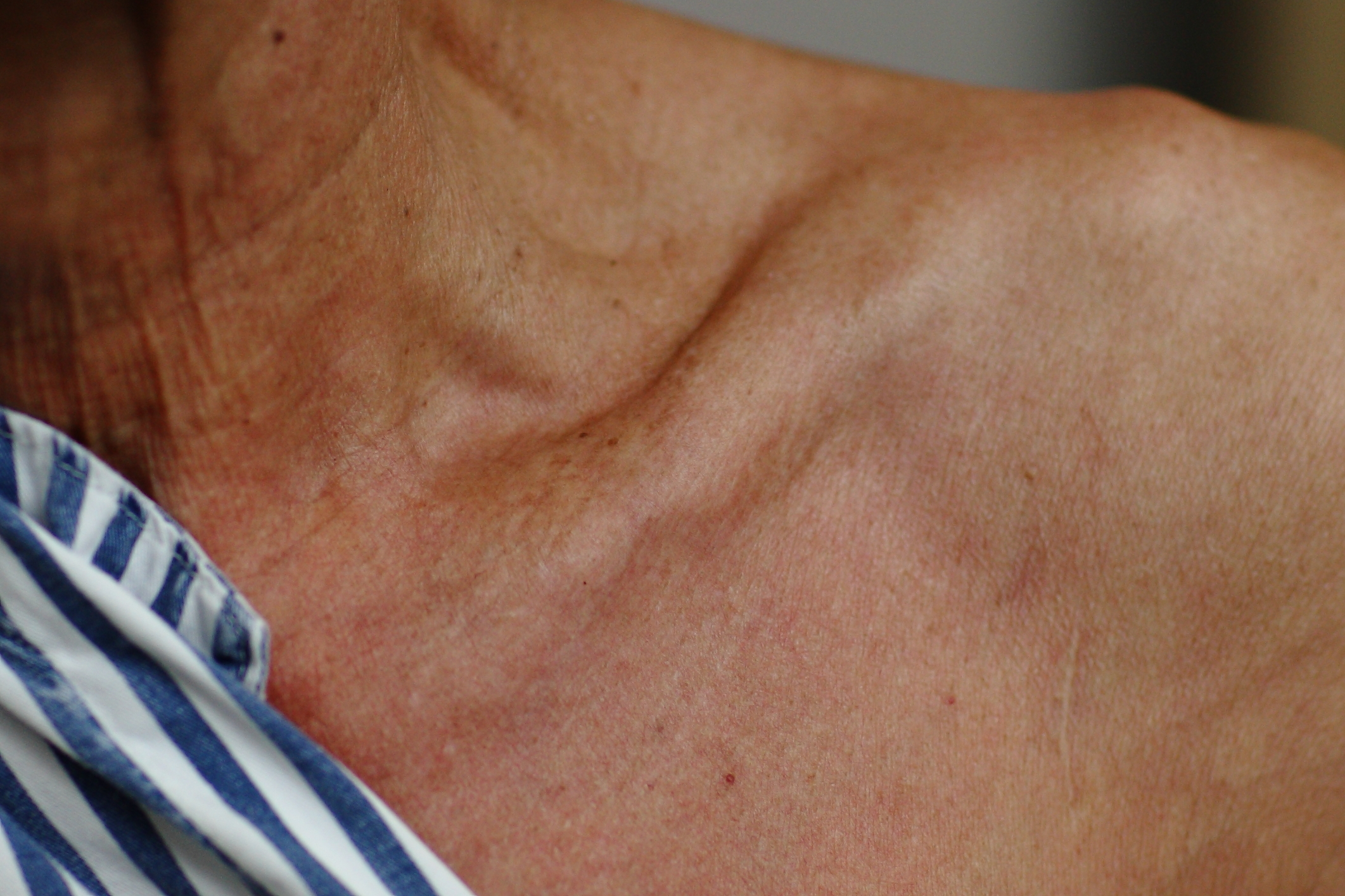 | 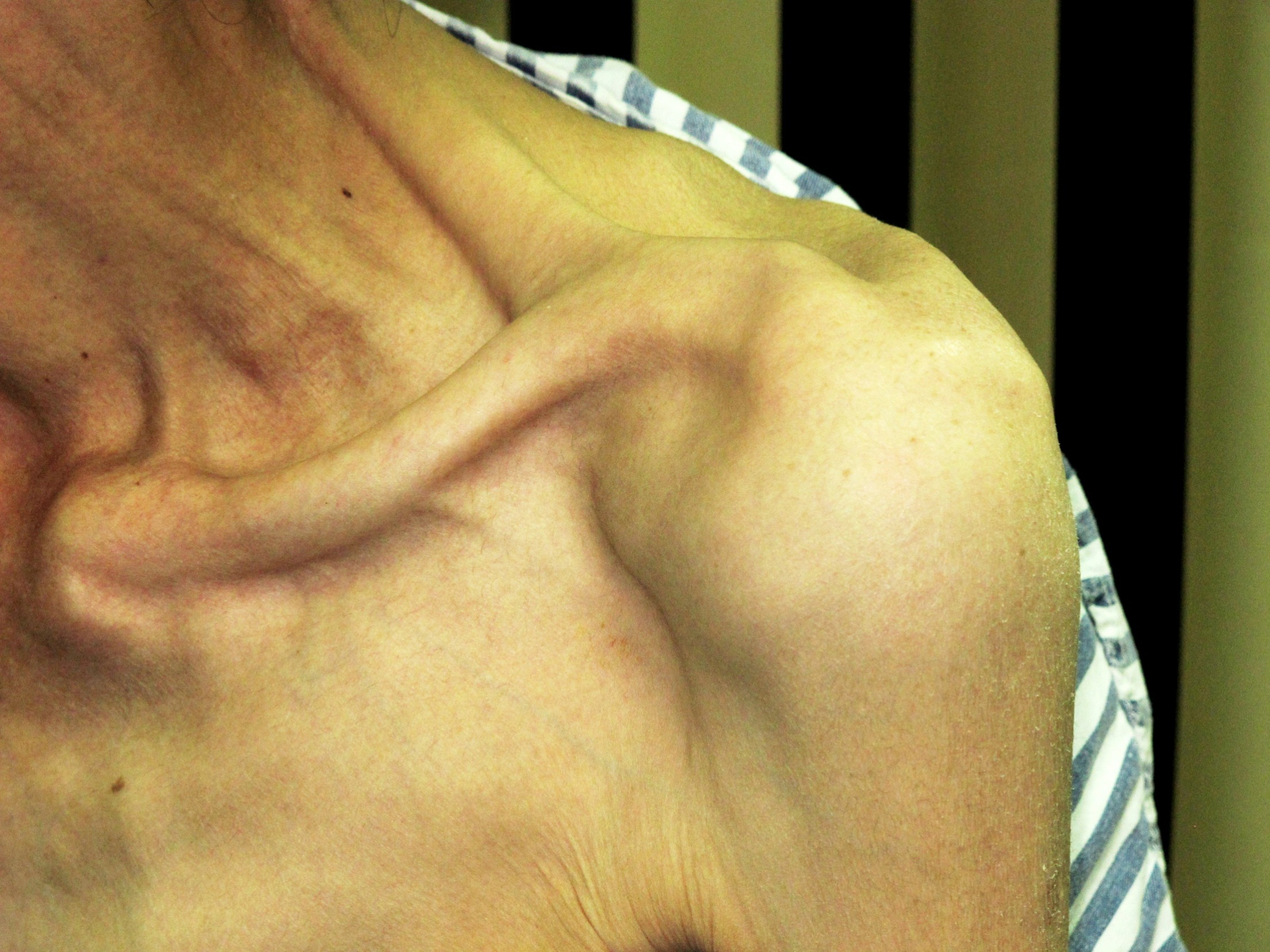 |
| Shoulder (deltoid) | Look for bulging shoulders, shape, drooping hands | Round | Slightly protruding shoulder crest | In-between | Squared shoulder lock joint with protruding bones |
|  |  | 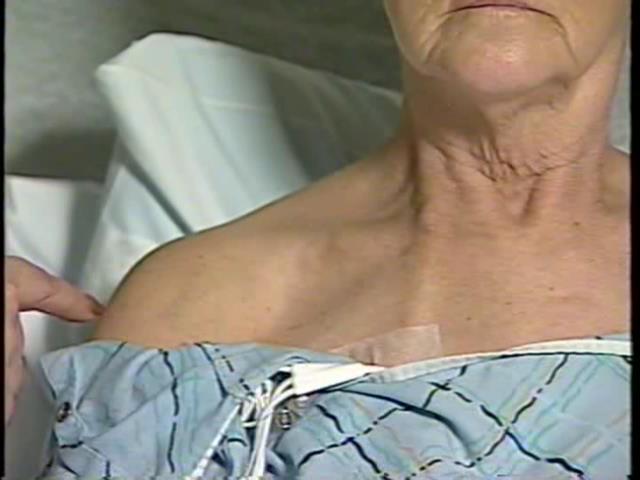 | 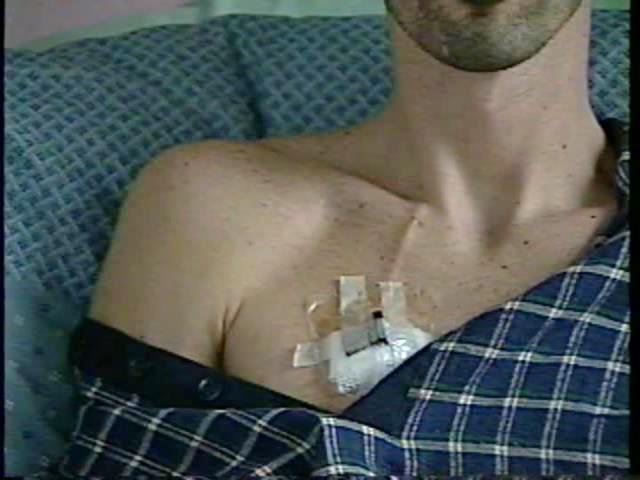 | 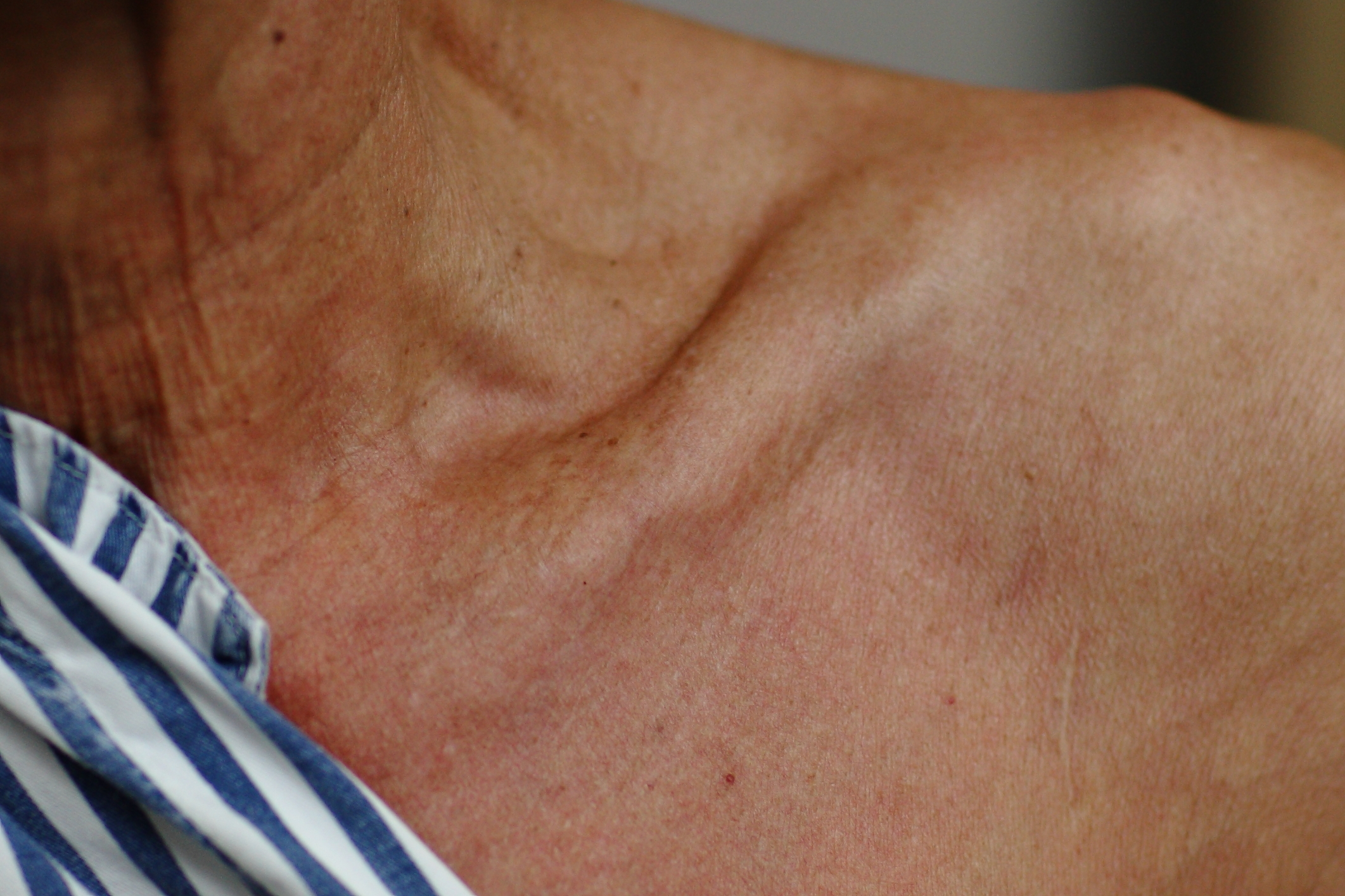 | 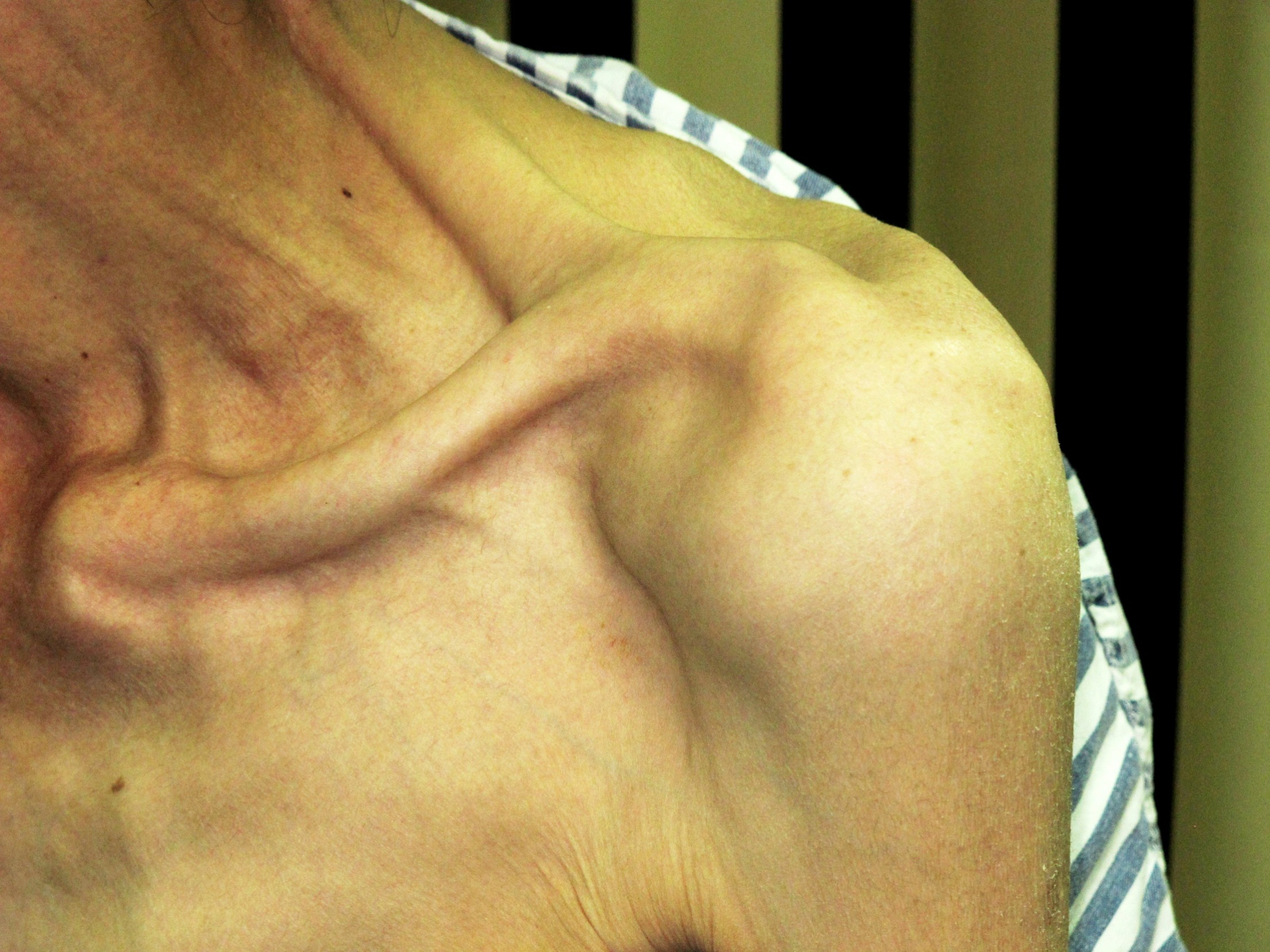 |
| Interosseous muscle | Observe the back of the hand, pinch the thumb and index finger against each other and observe if the tiger is sunken in | Muscles protrude when thumb and index finger are pinched against each other; women can flatten | Flat | Flats and depressions | Visible depression |
|  |  | 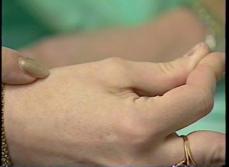 | 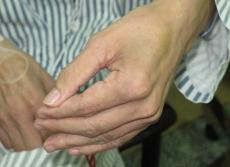 | 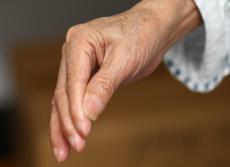 | 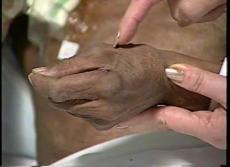 |
| Scapulae (latissimus dorsi, rhomboids, deltoids) | The patient pushes forward with both hands to see if the scapula protrudes | Scapulae not protruding, scapulae not depressed medially | Mild scapular projection, mild concavity between ribs, scapula, shoulder and spine | Scapula protruding, ribs, scapula, shoulder, interspinal recess | Scapulae markedly protruding, with significant depression between ribs, scapulae, shoulders and spine |
|  |  | 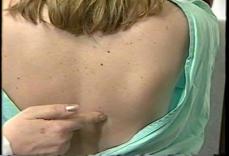 | 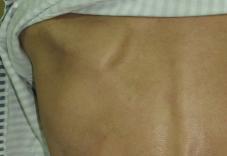 | 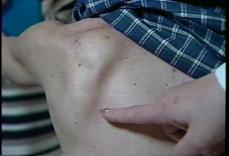 | 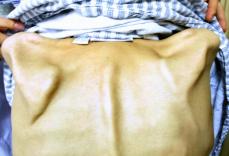 |
| Thigh (quadriceps) | Not as sensitive as upper limbs | Rounded, with pronounced tension | Mild wasting and weak muscle strength | In-between | markedly wasted thighs with little or no muscle tone |
|  |  | 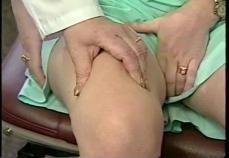 | 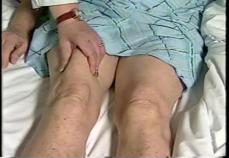 | 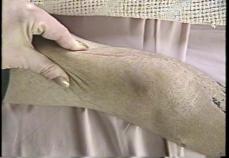 | 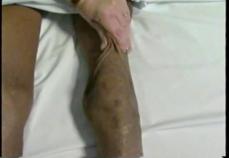 |
| Calf (gastrocnemius) |  | Muscular | Thin, with a muscular profile | Thin, with blurred muscle definition | Thin, no muscle definition, flabby and weak muscles |
|  |  | 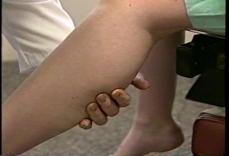 | 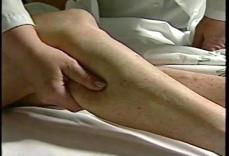 | 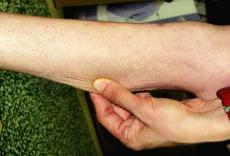 | 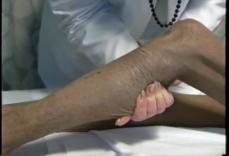 |
| Total muscle wasting score |  |  |  |  |  |
| ^1^ Assessment: The total score for this item is recorded for the muscle component only, and the score with the highest number of occurrences of "evaluation of muscle loss" is counted as the total score for this item. For example, four of the seven muscle scores are 2 and three are 3, giving an overall score of "2". | | | | | |
