# Supplementary material for: Associations of intermuscular adipose tissue and total muscle wasting score in PG-SGA with low muscle radiodensity and mass in nonmetastatic colorectal cancer: A two-center cohort study
Source: Front Nutr. 2022 Aug 25;9:967902. doi: 10.3389/fnut.2022.967902 (PMC9452825; doi:10.3389/fnut.2022.967902)
Supplement: Supplementary Table 2 — Area under the curve (AUC) for preoperative predictive factors in the training and validation cohort. [file Table_2.docx]

**Supplementary Table 2 |** **Area under the curve (AUC) for preoperative predictive factors in the training and validation cohort^1^.**

|  | Training cohort | | External validation cohort | |
| --- | --- | --- | --- | --- |
| Factors | LSMD(95% CI) | LSMI(95% CI) | LSMD(95% CI) | LSMI(95% CI) |
| Age, year | 0.661(0.631-0.690) | 0.743(0.715,0.770) | 0.619(0.580,0.657) | 0.633(0.594,0.671) |
| NLR | - | 0.780(0.753,0.805) | - | 0.645(0.606,0.682) |
| TNM stage | 0.624(0.593-0.654) | - | 0.658(0.619,0.695) | - |
| BMI, kg/m^2^ | 0.793(0.766,0.817) | 0.766(0.738,0.792) | 0.761(0.726,0.794) | 0.756(0.721,0.789) |
| Handgrip strength, kg | - | 0.681(0.652,0.710) | - | 0.586(0.546,0.624) |
| CC, cm | - | 0.646(0.615,0.675) | - | 0.650(0.611,0.687) |
| Walking speed, m/s | 0.712(0.683,0.740) | 0.698(0.669,0.727) | 0.665(0.626,0.701) | 0.567(0.527,0.606) |
| PEF, L/s | - | 0.669(0.639,0.698) | - | 0.644(0.606,0.682) |
| IMAT, cm^2^ | 0.619(0.588,0.649) | - | 0.574(0.535,0.613) |  |
| Total muscle wasting score | 0.748(0.720,0.774) | 0.772(0.745,0.798) | 0.638(0.599,0.675) | 0.659(0.620,0.696) |
| NRS-2002 score | 0.767(0.739,0.792) | 0.711(0.682,0.739) | 0.674(0.636,0.711) | 0.628(0.589,0.666) |
| Nomogram | 0.890(0.875,0.908) | 0.916(0.897,0.933) | 0.859(0.831,0.886) | 0.843(0.813,0.871) |
| 1 Data are analyzed using ROC curves. AUC, area under the curve; ROC, receiver operating characteristic; NLR, neutrophil-lymphocyte ratio; BMI, body mass index; CC, Calf circumference; PEF, Peak expiratory flow; IMAT, intermuscular adipose tissue; LSMD, low skeletal muscle radiodensity; LSMI, low skeletal muscle mass index. | | | | |
