# Supplementary material for: Associations of intermuscular adipose tissue and total muscle wasting score in PG-SGA with low muscle radiodensity and mass in nonmetastatic colorectal cancer: A two-center cohort study
Source: Front Nutr. 2022 Aug 25;9:967902. doi: 10.3389/fnut.2022.967902 (PMC9452825; doi:10.3389/fnut.2022.967902)
Supplement: Supplementary Table 3 — Univariate and multivariate cox regression analyses for overall survival (OS) in the training cohort. [file Table_3.docx]

**Supplementary Table 3 | Univariate and multivariate cox regression analyses for overall survival (OS) in the training cohort^1^.**

|  | Univariate | |  | Multivariate | |
| --- | --- | --- | --- | --- | --- |
| characteristics | HR (95% CI) | P value |  | HR (95% CI) | P value |
| Demographics |  |  |  |  |  |
| Sex | 0.95(0.78,1.17) | 0.633 |  |  |  |
| Age | 1.02(1.01,1.03) | <0.001 |  | 0.99(0.98,1.01) | 0.178 |
| Diabetes, n(%) | 0.71(0.53,0.96) | 0.024 |  | 0.82(0.61,1.10) | 0.184 |
| Alcohol, n(%) | 1.01(0.77,1.33) | 0.950 |  |  |  |
| Smoking history, n (%) | 0.94(0.73,1.20) | 0.616 |  |  |  |
| Tea drinking, n (%) | 1.06(0.76,1.49) | 0.724 |  |  |  |
| Weight loss, n (%) |  | 0.191 |  |  |  |
| Stable | Reference |  |  |  |  |
| 0–4.9% | 1.09(0.88,1.37) |  |  |  |  |
| ≥5% | 1.33(0.97,1.82) |  |  |  |  |
| Cancer stage, n (%) |  | <0.001 |  |  | <0.001 |
| I | Reference |  |  | Reference |  |
| II | 2.07(1.32,3.25) |  |  | 1.96(1.24,3.10) |  |
| III | 3.95(2.60,6.00) |  |  | 3.24(2.12,4.95) |  |
| Haematological Biomarkers |  |  |  |  |  |
| Creatinine, mg/dL | 1.00(0.99,1.01) | 0.353 |  |  |  |
| Hemoglobin, g/L | 0.99(0.99,1.01) | 0.899 |  |  |  |
| Prealbumin, mg/L | 1.00(1.00,1.01) | 0.654 |  |  |  |
| Albumin, g/L | 0.99(0.98,1.01) | 0.272 |  |  |  |
| NLR | 1.08(1.04,1.11) | <0.001 |  | 1.02(0.99,1.05) | 0.489 |
| CRP | 1.00(0.99,1.00) | 0.449 |  |  |  |
| Anthropometric measurements |  |  |  |  |  |
| BMI, kg/m^2^ | 0.93(0.91,0.96) | <0.001 |  | 1.02(0.99,1.05) | 0.258 |
| Handgrip strength, kg | 0.98(0.96,0.99) | 0.001 |  | 1.00(0.98,1.01) | 0.638 |
| MUAC, cm | 0.99(0.96,1.02) | 0.599 |  |  |  |
| TSF, mm | 1.00(0.98,1.01) | 0.917 |  |  |  |
| MAMC, cm | 0.97(0.94,1.02) | 0.994 |  |  |  |
| CC, cm | 0.98(0.96,1.01) | 0.079 |  |  |  |
| Walking speed m/s | 0.71(0.60,0.83) | <0.001 |  | 1.04(0.87,1.24) | 0.703 |
| Pulmonary Function |  |  |  |  |  |
| PEF, L/s | 0.92(0.85,0.99) | 0.036 |  | 0.96(0.88,1.04) | 0.314 |
| FEV1, L | 1.03(0.83,1.28) | 0.881 |  |  |  |
| VC, L | 0.78(0.74,1.06) | 0.201 |  |  |  |
| Body composition |  |  |  |  |  |
| IMAT, cm^2^ | 1.01(0.99,1.03) | 0.218 |  |  |  |
| VAT, cm^2^ | 1.00(0.99,1.01) | 0.322 |  |  |  |
| SAT, cm^2^ | 1.00(0.99,1.01) | 0.546 |  |  |  |
| VAT/SAT | 1.05(0.90,1.22) | 0.527 |  |  |  |
| LSMD | 2.94(2.39,3.60) | <0.001 |  | 2.10(1.61,2.78) | <0.001 |
| LSMI | 3.05(2.47,3.75) | <0.001 |  | 2.31(1.72,3.12) | <0.001 |
| Scores |  |  |  |  |  |
| Total muscle wasting score, n (%) |  | <0.001 |  |  | 0.061 |
| 0 | Reference |  |  | Reference |  |
| 1 | 1.24(0.89,1.71) |  |  | 0.87(0.62,1.23) |  |
| 2 | 2.39(1.82,3.14) |  |  | 1.32(0.97,1.81) |  |
| 3 | 2.39(1.78,3.16) |  |  | 1.15(0.82,1.62) |  |
| NRS-2002 score , n (%) |  | <0.001 |  |  | 0.102 |
| <3 | Reference |  |  | Reference |  |
| ≥3 | 1.78(1.46,2.19) |  |  | 0.78(0.57,1.05) |  |
| QLQ-C30 score | 1.00(0.99,1.01) | 0.374 |  |  |  |
| ^1^ Data are analyzed by univariate and multivariate Cox proportional hazards regression analysis. Risk factors with significance in univariate cox proportional hazard analysis were included in the multivariate cox proportional hazard analysis (p < 0.05). NLR, neutrophil-lymphocyte ratio; CRP: C-reactive Protein; BMI, body mass index; MUAC, mid-upper arm circumference; TSF, triceps skinfold thickness; MAMC, mid-arm muscle circumference; CC, Calf circumference; PEF, Peak expiratory flow; FEV1, Forced Expiratory Volume In 1s; VC, Vital Capacity; IMAT, intermuscular adipose tissue; VAT, visceral adipose tissue; SAT, subcutaneous adipose tissue; HU, Hounsfield unit; SMD, skeletal muscle radiodensity; SMI, skeletal muscle index; LSMI, low skeletal muscle mass index; LSMD, low skeletal muscle radiodensity; NRS, nutritional risk screening; QLQ-C30, Quality of Life Questionnare-Core 30. | | | | | |
